# Design of Ultra-Stable Solid Amine Adsorbents and Mechanisms of Hydroxyl Group-Dependent Deactivation for Reversible CO_2_ Capture from Flue Gas

**DOI:** 10.1007/s40820-025-01664-w

**Published:** 2025-02-28

**Authors:** Meng Zhao, Liang Huang, Yanshan Gao, Ziling Wang, Shuyu Liang, Xuancan Zhu, Qiang Wang, Hong He, Dermot O’Hare

**Affiliations:** 1 College of Environmental Science and Engineering, Beijing Forestry University, Beijing, 100083 People’s Republic of China; 2 State Key Laboratory of Efficient Production of Forest Resources, Beijing Forestry University, Beijing, 100083 People’s Republic of China; 3 Research Center of Solar Power and Refrigeration, Institute of Refrigeration and Cryogenics, Shanghai Jiao Tong University, Shanghai, 200240 People’s Republic of China; 4 State Key Joint Laboratory of Environment Simulation and Pollution Control, Research Centre for Eco-Environmental Sciences, Chinese Academy of Sciences, Beijing, 100085 People’s Republic of China; 5 Chemistry Research Laboratory, Department of Chemistry, University of Oxford, Mansfield Road, Oxford, OX1 3TA UK

**Keywords:** CO_2_ capture, Solid amine adsorbent, Long-term stability, Oxidative degradation, Urea formation

## Abstract

Although supported solid amine adsorbents have attracted great attention for CO_2_ capture, critical chemical deactivation problems including oxidative degradation and urea formation have severely restricted their practical applications for flue gas CO_2_ capture. In this work, we reveal that the nature of surface hydroxyl groups (metal hydroxyl Al–OH and nonmetal hydroxyl Si–OH) plays a key role in the deactivation mechanisms. The polyethyleneimine (PEI) supported on Al–OH-containing substrates suffers from severe oxidative degradation during the CO_2_ capture step due to the breakage of amine-support hydrogen bonding networks, but exhibits an excellent anti-urea formation feature by preventing dehydration of carbamate products under a pure CO_2_ regeneration atmosphere. In contrast, PEI supported on Si–OH-containing substrates exhibits excellent anti-oxidative stability under simulated flue gas conditions by forming a robust hydrogen bonding protective network with Si–OH, but suffers from obvious urea formation during the pure CO_2_ regeneration step. We also reveal that the urea formation problem for PEI-SBA-15 can be avoided by the incorporation of an OH-containing PEG additive. Based on the intrinsic understanding of degradation mechanisms, we successfully synthesized an adsorbent 40PEI-20PEG-SBA-15 that demonstrates outstanding stability and retention of a high CO_2_ capacity of 2.45 mmol g^−1^ over 1000 adsorption–desorption cycles, together with negligible capacity loss during aging in simulated flue gas (10% CO_2_ + 5% O_2_ + 3% H_2_O) for one month at 60–70 °C. We believe this work makes great contribution to the advancement in the field of ultra-stable solid amine-based CO_2_ capture materials.

**Supplementary Information:**

The online version contains supplementary material available at 10.1007/s40820-025-01664-w.

## Highlights

- We reveal that the nature of the hydrogen bonding networks formed by surface hydroxyl groups plays a key role in the deactivation mechanisms of supported polyethylenimine (PEI), which exhibits contrasting oxidative and anti-urea properties when supported on Al–OH- and Si–OH-containing substrates.
- PEG modification helps reduce urea formation for PEI supported on Si–OH-containing substrates, but does not prevent oxidation of the Al–OH-containing support. The resulted ultra-stable 40PEI-20PEG-SBA-15 showing outstanding stability over 1000 adsorption–desorption cycles (2.45 mmol g^−1^) and negligible capacity loss after one month in simulated flue gas.

**Supplementary Information:**

The online version contains supplementary material available at 10.1007/s40820-025-01664-w.

## Graphical abstract

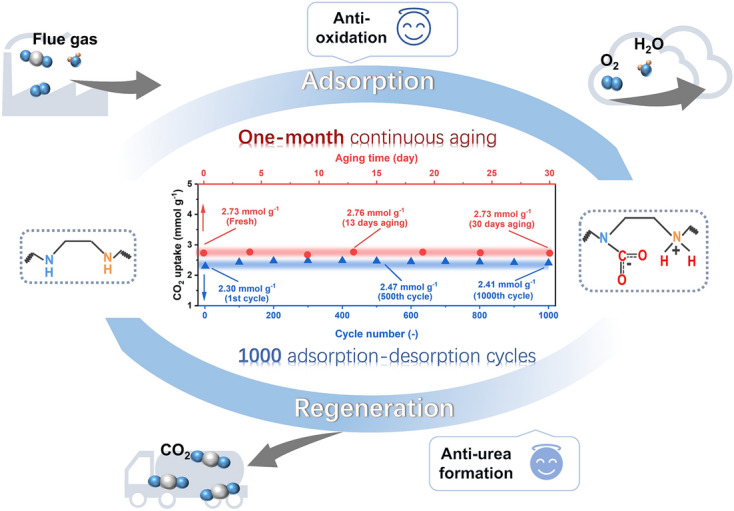

## Introduction

Post-combustion carbon dioxide (CO_2_) capture, which can be retrofitted into existing industrial facilities, is a “must-have” technology to both combat climate change and drive toward a net zero society [1–5]. The use of supported solid amine adsorbents for CO_2_ capture from actual flue gas streams of large point sources is a promising technology [6, 7]. However, this huge commercial potential will only be realized if the long-term cycling stability issue can be solved. During the CO_2_ adsorption and desorption operations, supported amines exposed to complex atmospheres (O_2_/CO_2_/H_2_O/N_2_) and varied temperatures (60–150 °C) are susceptible to degradation through multiple chemical pathways, including oxidative deactivation during CO_2_ capture period and urea formation during CO_2_-rich regeneration period [8]. Currently, most literature reports generally focus on one certain deactivation issue, lack of systematic evaluation under various conditions. However, for practical applications, a satisfied adsorbent should prevent both oxidative degradation and urea formation. Under certain conditions, the influence of H_2_O in the flue gas also needs to be considered. Therefore, mechanistic insights into different deactivation processes are highly demanded, which is essential for the design of ultra-stable supported solid amine adsorbents.

It is widely recognized that the interaction between amine and support is crucial for the performance and lifetime of solid amine adsorbents. Thus, to address the above-mentioned deactivation drawbacks, substantial efforts have been devoted to the exploration of appropriate substrates such as silica and alumina [9–12]. Early studies predominantly employed porous silica supports [13], but it was not considered as a good substrate due to the severe oxidative degradation of supported amines and the urea formation issue during the regeneration period [14, 15]. Sayari et al. [16] examined the stability of PEI-SiO_2_ adsorbent and found that the oxidative degradation problem could be relieved by the copresence of CO_2_ and O_2_. In the presence of CO_2_, amine groups are protected from oxygen attack, presumably because of their rapid conversion to carbamate and bicarbonate. Recently, Al_2_O_3_ has attracted much attention due to its excellent anti-urea formation property. Yan et al. [17, 18] found that the PEI-Al_2_O_3_ possesses significantly enhanced urea formation resistance over PEI-SiO_2_, which can be attributed to the selective conversion of primary amines to secondary amines by a cross-linking reaction between the amines and the support. However, Jones et al. [19] found that the PEI-Al_2_O_3_ adsorbent has a vital oxidative deactivation problem in the copresence of CO_2_ and O_2_ (0.04% CO_2_/21% O_2_) in both dry and humid streams, with obvious losses in stability (dry: 29%, humid: 52%) after 30 cycles. It is apparent that the substrates may exhibit good stability under one condition, but suffer from severe deactivation under the other. The reasons why different supports can lead to such significant differences in stability performance still remain a mystery. Therefore, revealing the key features of supports and their intrinsic influence on the deactivation mechanisms has now become critically important for the design of ultra-stable CO_2_ adsorbents.

In addition to developing more suitable supports, many studies found that the addition of additives such as polyethylene glycol (PEG) can improve the CO_2_ capture capacity and kinetics of solid amine-based adsorbents [20–25]. The positive effect of PEG was attributed to the formation of hydrogen bonding between -NH_2_ of amines and –OH groups of PEG, which increases the dispersion and amine efficiency. Chuang et al. [21] revealed for silica support adsorbent (TEPA-PEG-silica), such hydrogen bonding interactions can slow down the amine oxidation by blocking the amine sites from access to oxygen. However, the influence of PEG additive on two remaining vital issues is unexplored. Specifically, it is still unclear whether the incorporation of PEG can avoid both (i) the oxidative deactivation in the copresence of CO_2_ and O_2_ when Al_2_O_3_ was employed as support, and (ii) the urea formation during the regeneration period when silica was employed as support. In addition, most early studies on the incorporation of PEG were conducted in dry conditions. The adsorption of H_2_O may influence the CO_2_ capture capacity and result in excessive energy consumption for adsorbent regeneration [26–30]. Therefore, the PEG-induced improvements in the presence of H_2_O should also be clarified.

In this study, mechanistic insights into the oxidation degradation and CO_2_-induced urea formation deactivation were explored using PEI-based adsorbents supported on two different types of substrates. Specifically, Mg_*x*_Al hydroxide substrate that contains surface metal hydroxyls (e.g., Mg/Al–OH) and SBA-15 that features surface nonmetal hydroxyls (e.g., Si–OH) were employed. The influence of surface hydroxyl groups on the deactivation mechanisms of supported amines under different atmospheres (O_2_, CO_2_, CO_2_ + O_2_, CO_2_ + O_2_ + H_2_O) was comparatively examined. Interestingly, we revealed that the PEI supported on Al–OH-containing substrates suffers from severe oxidative degradation during the CO_2_ capture step, but exhibits enhanced anti-urea formation properties under the pure CO_2_ regeneration step. In contrast, the PEI supported on Si–OH-containing substrates exhibits excellent anti-oxidative stability, but suffers from obvious urea formation during the pure CO_2_ regeneration step. For the first time, we revealed that the urea formation problem of PEI-SBA-15 adsorbent could be relieved by the incorporation of OH-containing PEG. The influence of PEG additives on the CO_2_ adsorption/desorption kinetics and H_2_O co-adsorption was also investigated. More importantly, we designed an adsorbent 40PEI-20PEG-SBA-15 that demonstrates outstanding stability and retention of a high CO_2_ capacity of 2.45 mmol g^−1^ over 1000 adsorption–desorption cycles, along with negligible capacity loss during aging in simulated flue gas (10% CO_2_ + 5% O_2_ + 3% H_2_O) for one month at 60–70 °C. By such a tailored design of the adsorbent system, both the oxidative degradation problem in the presence of CO_2_ and O_2_ during the adsorption period and the urea formation problems during the pure CO_2_ regeneration step were solved. We believe this work could make a great contribution to the advancement of solid amine-based CO_2_ capture materials.

## Experimental Section

### Material Synthesis

The Mg_0.55_Al (*y*) nanosheets were prepared using a co-precipitation method, followed by aqueous miscible organic solvent treatment (AMOST). Typically, 50 mL of Mg–Al precursor solution containing 4.549 g Mg(NO_3_)_2_·6H_2_O and 12.101 g Al(NO_3_)_3_·9H_2_O was evenly added to an equal amount of basic solution containing 2.650 g NaCO_3_ under vigorous stirring. During the synthesis, the pH of the mixture was maintained at 10 by drop-wise addition of 4 M NaOH solution. The resulting mixture was then aged at 25 °C for another 12 h. After aging, the sediment was filtered, washed with deionized water to pH = 7, and further thoroughly rinsed with ethanol. The ethanol-washed solids were redispersed in ethanol and then stirred at 25 °C for at least 2 h. The product was finally collected by filtration, followed by vacuum drying at 60 °C overnight. The resulting samples were marked as Mg_0.55_Al (60 °C).

Preparation of the 60PEI-Mg_0.55_Al (60 °C) and 60PEI/SBA-15 adsorbents was using a common wet impregnation method. Typically, a certain amount of branched PEI (Shanghai Meryer Chemical Technology Co., Ltd, MW 600, 99%) was dissolved in methanol (30 mL), with stirring (600 rpm) at 25 °C under N_2_ protection for 30 min. Then, 0.5 g of synthesized Mg_0.55_Al (60 °C) or SBA-15 (Nanjing XFNANO Materials Tech Co., Ltd) was added to the above-mixed solution and stirred for another 3 h under N_2_ protection. The methanol was then removed by rotatory evaporation followed by vacuum drying at 60 °C overnight. To validate the general applicability of the results, commonly used support materials, including SiO_2_ (from Zhengzhou AIKEMU Chemical Co., Ltd, 99.5%) and γ-Al_2_O_3_ (from Nanjing XFNANO Materials Tech Co., Ltd, 99.9%), were also used as supports. The resulting hybrid adsorbents were denoted as *x*PEI-support, where *x* represents the weight ratio of PEI in the material.

Functionalization of PEI with PEG (Beijing Mreda Tech Co., Ltd, average Mn 200) was carried out by directly adding different amounts of PEG to the mixed solution of PEI and methanol and stirring at 25 °C under N_2_ protection for 30 min. The PEI-PEG-SBA-15 hybrid composites were also synthesized by the above-mentioned wet impregnation method. The synthesized adsorbents were named *x*PEI-*y*PEG-SBA-15, where *x* and *y* represent the weight ratio of PEI and PEG in the adsorbents, respectively.

### Materials Characterization

The X-ray diffraction (XRD) analysis consisted of wide-angle diffraction tests for *x*PEI-*y*PEG-Mg_0.55_Al (60 °C) performed on a Shimadzu XRD-7000 diffractometer with Cu Kα radiation (5°–80°, 5° min^−1^) and small-angle diffraction tests for *x*PEI-*y*PEG-SBA-15 conducted on a Bruker D8 ADVANCE with Co Kα radiation (0.2°–5°, 1° min^−1^). The pore structure properties of the adsorbents were determined at 77 K using a Builder SSA-7000 apparatus, with the specific surface area and average pore diameter calculated by the Brunauer–Emmett–Teller (BET) method and the pore volume analyzed using the Barrett–Joyner–Halenda (BJH) method. Elemental analysis and X-ray photoelectron spectroscopy (XPS) were used to analyze the functional groups, amino state distribution, and surface compositions of the adsorbents. The carbon (C), hydrogen (H), and nitrogen (N) content (wt%) of the adsorbent during a one-month continuous aging experiment was analyzed using an Elementar Unicube elemental analyzer. X-ray photoelectron spectroscopy (XPS) analysis was performed using a Thermo Scientific Escalab 250Xi instrument with a monochromatic Al Kα radiation (1486.6 eV).

### CO_2_ Adsorption Tests

CO_2_ adsorption–desorption profiles and long-term cyclic stability data were collected using an in situ TGA analyzer (TA55 instrument). Initially, 10 mg of the sample was pretreated under N_2_ flow at a rate of 40 mL min^−1^, followed by switching from N_2_ to CO_2_ at the desired set temperature for adsorption. Cyclic breakthrough experiments were conducted in the presence of moisture utilizing a homemade transient gas-flow setup. Approximately 0.2 g of finely ground adsorbents was loaded into a quartz continuous stirred tank reactor (CSTR) micro-reactor. A small furnace (220 mm total length) was employed for heating, with the reactor positioned horizontally. Moisture for the inlet gases (3 vol%) was generated using a scrubbing bottle immersed in a thermostatic water bath. All tubing downstream of the scrubbing bottle is heated to uphold a temperature of 70 °C to prevent water condensation. CO_2_ adsorption was carried out at 70 °C with a feed rate of 40 mL min^−1^ consisting of 10% CO_2_, 5% O_2_, and 3 vol% H_2_O for 10 min, while desorption was performed at 70 °C in a flow of pure nitrogen (40 mL min^−1^) for 90 min. The CO_2_ concentration at the reactor outlet was continuously monitored online using a THA100S nondispersive infrared analyzer (0–20%). The CO_2_ uptake was calculated using Eq. 1 in the cyclic breakthrough experiments. Every breakthrough experiment was adjusted by subtracting the blank measurements.

1 $$q = \frac{{\mathop \smallint \nolimits_{0}^{t} \left( {C_{{{\text{CO}}_{2} ,{\text{in}}}} - C_{{{\text{CO}}_{2} ,{\text{out}}}} } \right)Q_{{{\text{in}}}} {\text{d}}t}}{m}$$

where $q$: CO_2_ uptakes (mmol g^−1^); $CO_{2}, in$ : CO_2_ concentration in the inlet stream (mmol m^−3^); $C_{{{\text{CO}}_{2} ,{\text{out}}}}$: CO_2_ concentration in the outlet stream (mmol m^−3^); ${Q}_{\text{in}}:$ Flow rate of the inlet stream (m^3^ min^−1^); $m$: Initial mass of the adsorbent (g).

### Oxidative Aging Experiments

For exploring the effect of supports on the oxidative stability of impregnated PEI under pure O_2_/CO_2_ + O_2_ conditions, oxidative aging experiments were performed using a thermogravimetric analyzer (TA55 instrument). Prior to oxidation, about 10 mg of materials was degassed at 120 °C for 1 h in a N_2_ gas stream. Oxidative aging was then performed by direct exposure of the adsorbents in flowing O_2_-containing gases (40 mL min^−1^) at a given temperature. The CO_2_ adsorption capacities of the fresh and oxidized adsorbents were also tested by TGA. The oxidative aging experiments, aimed at exploring the effect of supports on the oxidative stability of impregnated PEI in the presence of moisture, were conducted within the above-mentioned homemade transient gas-flow system. About 0.1 g sample underwent pretreatment in a CSTR micro-reactor under N_2_ flow at 40 mL min^−1^, maintained at 120 °C for 60 min. The oxidative experiments were performed at 120 °C with a feed (40 mL min^−1^) consisting of 5% O_2_, with and without 3 vol% H_2_O, respectively. The one-month continuous aging experiment of 40PEI-20PEG-SBA-15 was performed utilizing a laboratory-scale tube furnace. Following pretreatment under N_2_ flow for 1 h at 90 °C, the aging experiment proceeded at temperatures ranging from 60 to 70 °C, with a feed (40 mL min^−1^) consisting of 10% CO_2_, 5% O_2_, and 3 vol% H_2_O.

### In Situ DRIFTS Studies

In situ DRIFTS experiments were implemented on a PerkinElmer Spectrum 3 FTIR spectrometer (4 cm^−1^ resolution) equipped with an MCT detector to identify the formation of oxidized species and urea compounds on deactivated adsorbents. Approximately 50 mg of adsorbents was loaded into a temperature-controlled in situ DRIFTS reactor cell with a ZnSe window, and the DRIFTS spectra for analysis were obtained by subtracting the background acquired at the reaction temperature under N_2_ flow and then recorded under reaction conditions with the averaged spectrum of 128 scans from 700 to 4000 cm^−1^.

### Transient Oxidation Coupled with Mass Spectrometry Studies

Transient oxidation experiments to study the stabilization mechanism in the carbon-containing flue gas were conducted in a homemade transient gas-flow system described above. Approximately 0.3 g of adsorbents was first pretreated in an N_2_ flow of 40 mL min^−1^ at 120 °C for 1 h, followed by purging with either 5% O_2_ or a gas mixture of 10% CO_2_ + 5% O_2_, while the reactor temperature rose from 120 to 220 °C with a heating rate of 1 °C min^−1^. The signals of the NH_3_ (m/z = 15), O_2_ (m/z = 32), and CO_2_ (m/z = 44) from the outlet emissions were recorded by an online mass spectrometer (Hiden Analytical, HAS 301). While the mass-to-charge ratio (m/z = 44) could also potentially correspond to N_2_O, trace amounts of N_2_O may form as a byproduct of the oxidation process alongside NH_3_. However, given its low concentration, N_2_O is unlikely to significantly impact the conclusions of the study [31]. Therefore, in this work, we attribute the m/z = 44 signal primarily to CO_2_.

## Results and Discussion

### Comparative Cyclic Stability Evaluation under Different Conditions

In order to reveal the key factors of supports that influence the deactivation mechanisms of supported amines, two different types of substrates including Mg_*x*_Al hydroxide substrate that contains surface metal hydroxyls (e.g., Mg/Al–OH) and SBA-15 that features surface nonmetal hydroxyls (e.g., Si–OH) were employed. Both supports were impregnated with PEI or PEI/PEG mixtures to make *x*PEI-*y*PEG-Mg_0.55_Al (60 °C) and *x*PEI-*y*PEG-SBA-15, where *x* and *y* represent the weight ratio of PEI and PEG in the adsorbent, respectively. N_2_ adsorption–desorption isotherms for both SBA-15 and Mg_0.55_Al (60 °C) exhibit typical type IV profiles with hysteresis loops, confirming their well-defined mesoporous structures (Fig. S1). After functionalization with PEI or PEI/PEG mixtures, significant reductions in BET surface area and pore volume were observed, consistent with the successful incorporation of amine groups into the pore channels of both materials (Tables S1 and S2).

XRD analyses further elucidate the structural changes in these materials (Fig. S2). For Mg_0.55_Al (60 °C), the pristine sample exhibits characteristic diffraction peaks corresponding to layered double hydroxides (LDHs) [32]. Upon the incorporation of PEI and PEG, the LDH layered structure remains intact, with the (003) and (006) peaks shifting slightly to lower 2θ values, suggesting a modest increase in the interlayer spacing due to the loading of PEI/PEG [33, 34]. For SBA-15, the pristine material shows well-defined low-angle diffraction peaks at (100), (110), and (200), which correspond to its ordered hexagonal mesoporous structure. After the incorporation of PEI and PEG, the intensity of the (100) peak is significantly reduced, while the higher-order reflections (110) and (200) become increasingly indistinct, indicative of pore-filling effects [35].

The cyclic stability of two representative adsorbents 60PEI-Mg_0.55_Al (60 °C) and 60PEI-SBA-15 was comparatively investigated using three adsorption–desorption conditions, which were designed to mimic different operational scenarios. Condition (1): adsorption in 10% CO_2_ for 10 min at 75 °C, and desorption in pure N_2_ for 15 min at 120 °C; condition (2): adsorption in 10% CO_2_ + 5% O_2_ for 10 min at 75 °C, and desorption in pure N_2_ for 15 min at 120 °C; and condition (3): adsorption in 100% CO_2_ for 2 min at 75 °C and desorption in 100% CO_2_ for 1 min at 165 °C. Condition 1 simulates a conventional flue gas adsorption–desorption cycle commonly used in laboratory studies; Condition 2 assesses the impact of O_2_ on adsorbent stability, possibly through oxidative degradation of the impregnated amines; and Condition 3 evaluates the regeneration process in pure CO_2_, where amines may undergo irreversible deactivation due to urea formation.

**Fig. 1:**
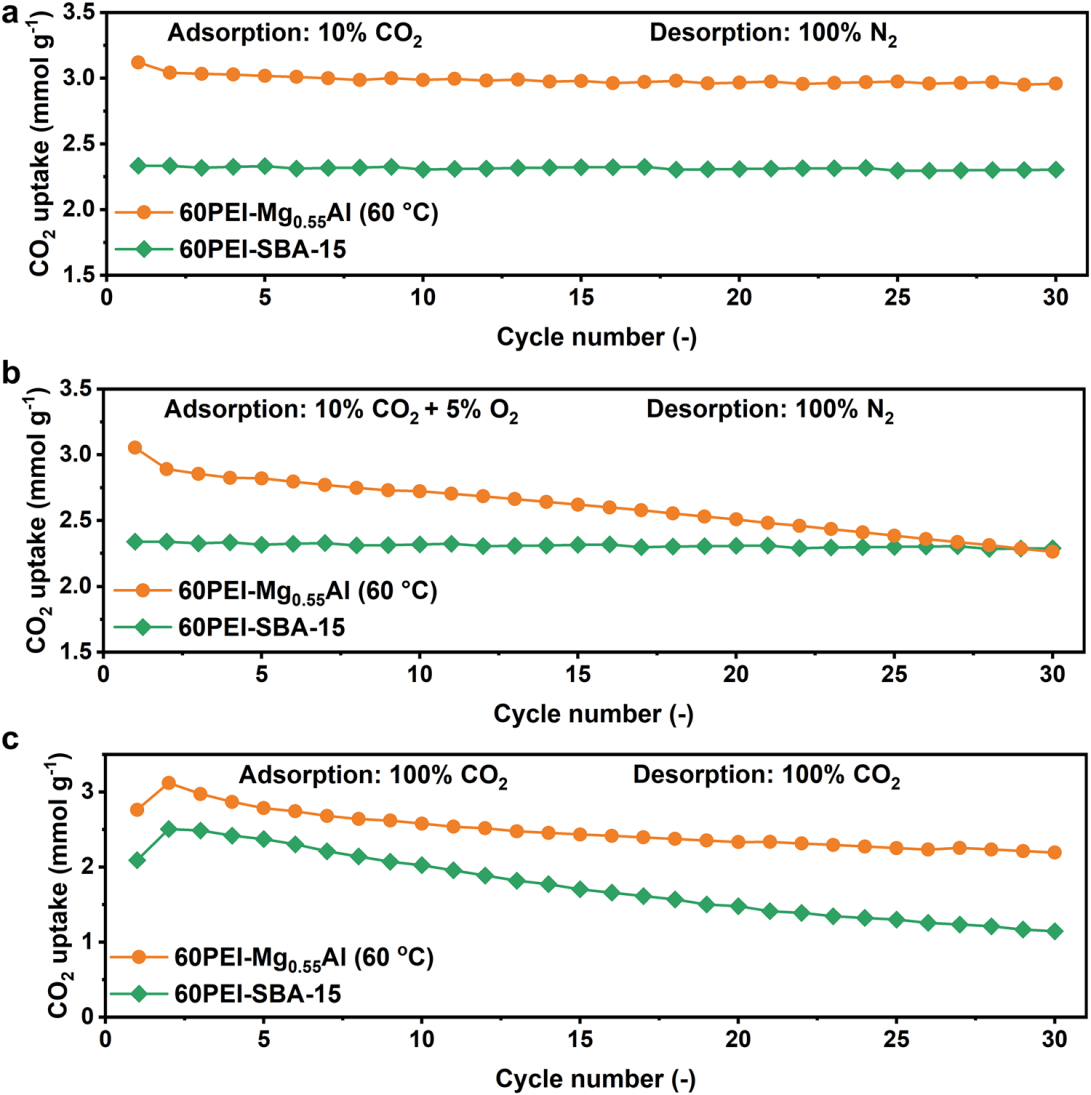
Cyclic CO_2_ adsorption performance. **a** CO_2_ uptakes of 60PEI-Mg_0.55_Al (60 °C) and 60PEI-SBA-15 over 30 cycles (adsorption at 75 °C in 10% CO_2_ for 10 min) and desorption at 120 °C in 100% N_2_ for 15 min). **b** CO_2_ uptakes of 60PEI-Mg_0.55_Al (60 °C) and 60PEI-SBA-15 over 30 cycles (adsorption at 75 °C in 10% CO_2_ + 5% O_2_ for 10 min and desorption at 120 °C in 100% N_2_ for 15 min). **c** CO_2_ uptakes of 60PEI-Mg_0.55_Al (60 °C) and 60PEI-SBA-15 over 30 cycles (adsorption at 75 °C in 100% CO_2_ for 2 min and desorption at 165 °C in 100% CO_2_ for 1 min)

Interestingly, we found that the cyclic stability of impregnated PEI depends on not only the supporting substrate but also the adsorption–desorption conditions (Fig. 1). In adsorption–desorption condition 1 (adsorption in 10% CO_2_ and desorption in pure N_2_), 60PEI-Mg_0.55_Al (60 °C) exhibited higher average CO_2_ uptake (3.00 mmol g^−1^) than 60PEI-SBA-15 (2.31 mmol g^−1^), and both samples maintained high stability (Fig. 1a). In adsorption–desorption condition 2 (adsorption in 10% CO_2_ + 5% O_2_ and desorption in pure N_2_), the CO_2_ uptake of 60PEI-Mg_0.55_Al (60 °C) irreversibly decreased, with a capacity loss of 25.94% after 30 cycles (Fig. 1b). In contrast, 60PEI-SBA-15 showed much better regenerability, with a slight capacity loss of 2.13% under the same condition. While in adsorption–desorption condition 3 (adsorption in 100% CO_2_ and desorption in 100% CO_2_), 60PEI-Mg_0.55_Al (60 °C) is relatively stable but 60PEI-SBA-15 showed a rapid decay and its CO_2_ uptake dropped from 2.09 to 1.14 mmol g^−1^ after 30 cycles (Fig. 1c). In any adsorption–desorption conditions, the deactivations might be due to either the oxidative degradation of amines during the adsorption period or the CO_2_-induced urea formation during the regeneration period, or the both, which are systematically investigated in detail in the following sections. Particularly the reason why the supporting substrates have such a significant influence on the cyclic stability is of great research interest. For practical applications, the supported amines are expected to experience more complex atmospheres (O_2_/CO_2_/H_2_O) and varied temperatures (60–150 °C), and have to overcome multiple chemical deactivations. Therefore, we believe a comprehensive understanding of the mechanistic insights into different deactivation processes is essential for the design of ultra-stable supported solid amine adsorbents.

### Oxidative Degradation Mechanism in Pure Oxygen

**Fig. 2:**
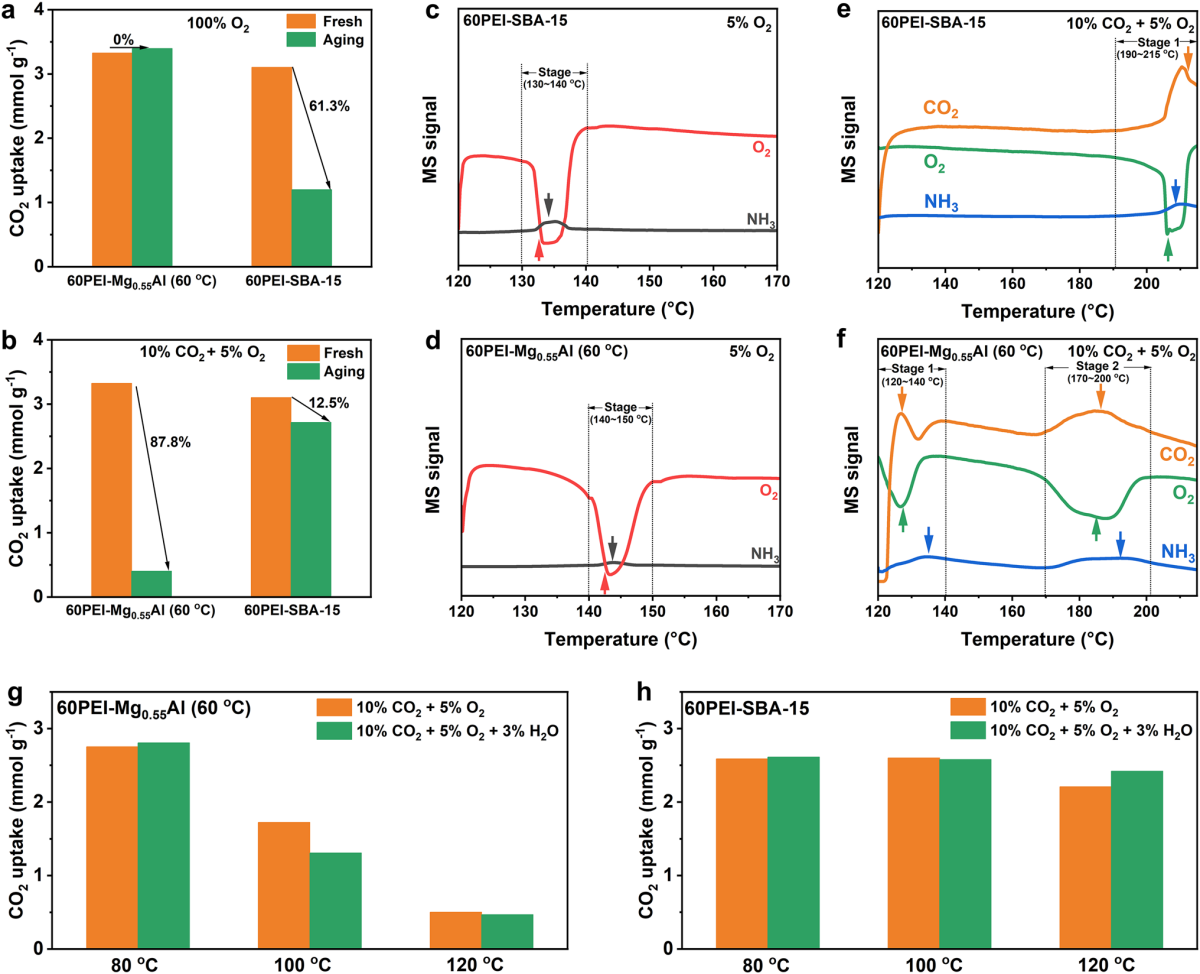
CO_2_ uptakes of 60PEI-Mg_0.55_Al (60 °C) and 60PEI-SBA-15 before and after aging treatment: **a** aged in pure O_2_ and **b** aged in 10% CO_2_ + 5% O_2_. The mass spectrometry (MS) signals of NH_3_ (m/z = 15) and O_2_ (m/z = 32) during the passage of 5% O_2_ over **c** 60PEI-SBA-15 and **d** 60PEI-Mg_0.55_Al (60 °C) samples. The MS signals of NH_3_ (m/z = 15), O_2_ (m/z = 32), and CO_2_ (m/z = 44) during the passage of 10% CO_2_ + 5% O_2_ over **e** 60PEI-SBA-15 and **f** 60PEI-Mg_0.55_Al (60 °C) samples. CO_2_ uptakes of **g** 60PEI-Mg_0.55_Al (60 °C) and **h** 60PEI-SBA-15 after aging treatment in 10% CO_2_ + 5% O_2_, both with and without 3 vol% H_2_O

The oxidative stability of PEI supported on Mg_0.55_Al and SBA-15 was comparatively studied by an aging experiment performed at 120 °C in pure O_2_ for 1 h. The CO_2_ uptakes of 60PEI-Mg_0.55_Al (60 °C) and 60PEI-SBA-15 before and after aging treatment were tested in 10% CO_2_ at 75 °C for 1 h. We were surprised to find that the aged 60PEI-Mg_0.55_Al (60 °C) showed almost no capacity loss, which indicates that its oxidation deactivation in O_2_ is negligible. While a significant capacity loss of 61.3% was observed with aged 60PEI-SBA-15, suggesting it underwent severe oxidative degradation during O_2_ aging treatment (Fig. 2a). The oxidative degradation of aged 60PEI-SBA-15 was further confirmed by in situ DRIFTS measurements under 100% O_2_ atmosphere at 120 °C. The typical PEI degradation products were clearly observed, with the appearance of the C=O/C=N stretching band of nonbasic amide/imine species at 1677 cm^−1^ and new primary amine sites in the PEI backbone at 1603 cm^−1^ (Fig. S3a, b) [21, 36]. In contrast, no such species were observed with aged 60PEI-Mg_0.55_Al (60 °C), confirming its higher oxidation resistance under the pure O_2_ aging condition.

Then, the interaction between impregnated PEI and inorganic support was investigated using XPS analyses, which is crucial for understanding the deactivation mechanism. As shown in Fig. S4, the O 1*s* spectrum can be deconvoluted into three subpeaks corresponding to H–O–H, M–OH, and O^2−^ species [37]. After PEI impregnation, the Al 2*p* and O 1*s* peaks of 60PEI-Mg_0.55_Al (60 °C), as well as the Si 2*p* and O 1*s* peak of 60PEI-SBA-15, shift to lower binding energies. This shift is attributed to hydrogen bonding between the amine groups (–NH or –NH_2_) in PEI and surface hydroxyl groups (Al–OH and Si–OH), which increases the electron cloud density of the support [37, 38]. Notably, the –OH peak area of Mg_0.55_Al (60 °C) significantly decreased compared to SBA-15, with the proportion of -OH dropping from 53.79% to 46.93%. This substantial reduction suggests the formation of strong hydrogen bonding between the surface hydroxyl groups (-OH) of Mg_0.55_Al (60 °C) and nitrogen atoms in PEI, which plays a key role in stabilizing the amine structure on the support. The basicity of surface -OH on Mg_0.55_Al (60 °C) is stronger than that on SBA-15, leading to a more robust hydrogen bonding network that protects amines from O_2_ access. This explains the reason why Mg_0.55_Al (60 °C) gives much better anti-oxidation performance than SBA-15 in pure O_2_.

### Oxidative Degradation Mechanism in Simulated Flue Gas

For practical application consideration, it is more important to evaluate the anti-oxidation property of the adsorbents under a simulated flue gas condition. Therefore, the oxidative stability of PEI supported on Mg_0.55_Al and SBA-15 was further comparatively studied by aging at 120 °C in 10% CO_2_ + 5% O_2_ for 1 h (Fig. 2b). Surprisingly, the 60PEI-Mg_0.55_Al (60 °C) became very vulnerable under this condition, with a significant CO_2_ uptake drop of 87.8%, much higher than that in pure O_2_ (0%). In contrast, the introduction of CO_2_ greatly mitigated the oxidative degradation of 60PEI-SBA-15, and its CO_2_ uptake drop of 12% is much less than that in pure O_2_ (61.3%). These results indicate the coexistence of CO_2_ and O_2_ could accelerate the oxidation of PEI supported on Mg_0.55_Al (60 °C) but retards its oxidation on SBA-15. This conclusion was confirmed by temperature-programmed oxidation (TPO) analyses. In 5% O_2_ flow, the O_2_ consumption for 60PEI-Mg_0.55_Al (60 °C) was primarily observed at 140–150 °C, slightly higher than that for 60PEI-SBA-15 (130–140 °C) (Fig. 2c, d). However, in 10% CO_2_ + 5% O_2_ flow, the O_2_ consumption peak for 60PEI-SBA-15 shifted to a much higher temperature (ca. 206 °C), indicating that the presence of CO_2_ retards the oxidation of PEI supported on SBA-15. Although a similar upward shift in the O_2_ consumption peak around 188 °C was observed with 60PEI-Mg_0.55_Al (60 °C), a new O_2_ consumption peak emerged at a significantly lower temperature of 126 °C, suggesting that at this temperature, the CO_2_ markedly accelerates the oxidation of PEI supported on Mg_0.55_Al (60 °C) (Fig. 2e, f). The significant drop in the O_2_ signal, accompanied by the generation of NH_3_, is primarily attributed to the further hydrolysis of oxidation products, leading to the cleavage of C–N bonds [15, 39]. In addition to the volatile gaseous products, we infer that nonbasic oxidation species remain attached to the Mg_0.55_Al (60 °C) support. This hypothesis is further corroborated by in situ DRIFTS analysis, which will be discussed in greater detail in the following sections. These results further confirm that the support significantly influences the oxidative degradation behavior of PEI. The influence of H_2_O, an essential component of flue gas, is also comparatively studied. Aging tests were conducted for 1 h at various temperatures (80, 100, and 120 °C) in a 10% CO_2_ + 5% O_2_ atmosphere, with and without water vapor. The CO_2_ uptake after aging was measured at 75 °C in a 10% CO_2_ atmosphere (Fig. 2g, h). It is apparent that the addition of 3% H_2_O did not change the degradation trends for both samples, with little influence on their CO_2_ uptakes.

To elucidate the oxidative degradation mechanism of these two samples in the copresence of CO_2_ and O_2_, in situ DRIFTS analyses were performed at temperatures from 80 to 150 °C. With 60PEI-Mg_0.55_Al (60 °C), as shown in Fig. S5, the amine stretching bands at 3408 and 3328 cm^−1^ (green region) emerged at 130 °C, and the intensity increased as the temperature rose. This observation clearly indicates that the disruption of hydrogen bonding in the 60PEI-Mg_0.55_Al (60 °C) system facilitated the return of amine molecules to a hydrogen bonding-free state [21]. Notably, particularly starting at 140 °C, the N–H deformation peak at 1652 cm^−1^ (blue region) exhibited a significant shift, while a distinct C=O/C=N stretching band corresponding to nonbasic amide/imine species appeared at 1673 cm^−1^ (orange region) [21, 40]. These findings suggest that the introduction of CO_2_ into the 60PEI-Mg_0.55_Al (60 °C) system disrupted the hydrogen bonding protective layer between PEI and Mg_0.55_Al (60 °C), consequently leading to a severe oxidation of PEI. This oxidation degradation ultimately results in the formation of nonbasic imines and amides, which remain attached to the support, accompanied by the release of volatile gaseous species such as NH_3_. Figure S6 presents the time-resolved DRIFTS spectra of 60PEI-SBA-15 under 10% CO_2_ + 5% O_2_ at 120 °C. Characteristic peaks of ammonium carbamates in the range of 1200–1700 cm^−1^ were observed. And no significant amide or imine oxidation products were detected. The peak at 1645 cm^−1^ corresponds to the N–H deformation vibration of ammonium ions (RNH_3_^+^), while the absorption bands at 1558 and 1500 cm^−1^ are attributed to the stretching vibrations of C=O in carbamate ions (NHCOO^–^). Additionally, the peaks at 1410 and 1319 cm^−1^ correspond to the C–N stretching vibration in NHCOO^–^ and the deformation vibration of the NCOO^–^ framework, respectively. Moreover, the enhancement of the C=O stretching band at 1706 cm^−1^ indicates the formation of carbamic acid (NCOOH) [40–43]. Furthermore, no stretching vibration bands of free, unbound amines were observed in the 3200–3500 cm^−1^ range for 60PEI-SBA-15, suggesting that, although the hydrogen bonding interactions between PEI and the nonmetal hydroxyl groups of Si–OH are relatively weak, the introduction of CO_2_ does not disrupt these interactions. Conversely, the mobility of the PEI chains is enhanced, allowing for rapid reaction with CO_2_ to form carbamate species with greater oxidative stability, thereby effectively inhibiting O_2_-induced oxidative degradation. While carbamate species were also detected on the surface of 60PEI-Mg_0.55_Al (60 °C), the breakage of amine-support hydrogen bonding networks emerged as the dominant factor, leading to the irreversible oxidative degradation of PEI.

**Fig. 3:**
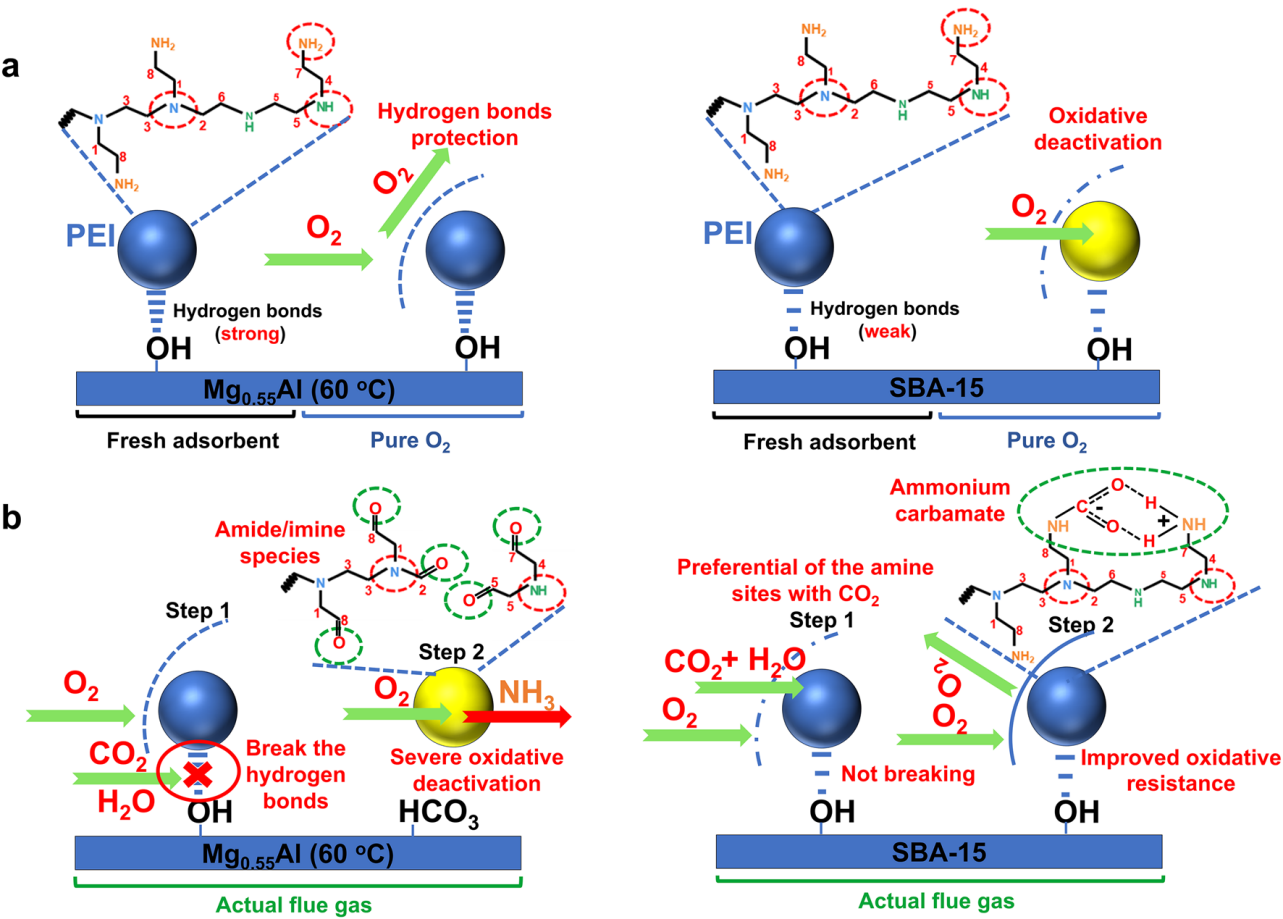
Illustration of the underlying oxidative degradation mechanisms. Oxidative degradation process under **a** pure O_2_ conditions and **b** actual flue gas conditions

Overall, our studies reveal that the properties of hydroxyl groups on the surface of supports—specifically, metal hydroxyl (Al–OH), and nonmetal hydroxyl (Si–OH)—play a crucial role in the oxidative deactivation mechanism. PEI supported on Mg_0.55_Al (60 °C) containing Al–OH groups demonstrates excellent oxidative stability under pure O_2_ conditions; however, it experiences significant oxidative degradation in simulated flue gas (10% CO_2_ + 5% O_2_ + 3% H_2_O) conditions. The Al–OH groups on Mg_0.55_Al (60 °C) effectively shield the NH_2_ and NH sites through strong hydrogen bonding interactions, stabilizing the PEI chains and inhibiting direct interaction between O_2_ and the NH_2_/NH sites, thereby protecting the amine sites from oxidation. Nevertheless, the introduction of concentrated CO_2_ disrupts the hydrogen bonding network between PEI and Al–OH. This disruption leads to the loss of the protective hydrogen bonding for the PEI chains, rendering them more susceptible to exposure to oxidative gases (Fig. 3a). Conversely, PEI loaded on SBA-15 containing Si–OH groups is susceptible to severe oxidative degradation under pure oxygen, but can maintain a high level of oxidative stability in simulated flue gas (10% CO_2_ + 5% O_2_ + 3% H_2_O) conditions. Although the hydrogen bonding network formed between the weakly basic Si–OH and PEI sites is relatively weak, this weak hydrogen bonding allows for increased flexibility of the PEI chains, facilitating more effective interactions with the external environment (such as CO_2_ and O_2_). Therefore, under pure oxygen, the active sites of PEI supported on SBA-15 are readily attacked by oxygen, leading to significant oxidative deactivation. However, in the copresence of CO_2_ and O_2_, this hydrogen bonding network exhibits greater stability and is not easily disrupted by CO_2_. The relatively flexible PEI polymer chains tend to rapidly react with CO_2_ to form carbamate species, thereby stabilizing the system and effectively inhibiting the approach of oxygen (Fig. 3b).

### Hydroxyl Group-Dependent Urea Formation Mechanism

The CO_2_-induced urea formation issue during the regeneration period is another important challenge for supported solid amine adsorbents. In this contribution, the influence of supporting substrates on the urea formation is studied. For PEI, the formation of cyclic urea species is triggered by direct intramolecular dehydration of ammonium carbamate [44–47]. Figure S7 shows the TG profiles of 60PEI-SBA-15 and 60PEI-Mg_0.55_Al (60 °C) in 100% N_2_ or 10% CO_2_ flow. The accumulation of thermally stable urea compounds via CO_2_-induced dehydration reactions tends to result in a weight gain [46]. Different from the TG curve obtained in N_2_ atmosphere, a weight gain of 1.67 wt% was observed over 60PEI-SBA-15 during continuous heating and CO_2_ exposure, indicating the formation of urea. While 60PEI-Mg_0.55_Al (60 °C) exhibited an opposite trend in 10% CO_2_ atmosphere. After the initial CO_2_ adsorption, an eventual weight loss of 1.77 wt% was observed, which can be attributed to the slight amine volatilization and the dehydroxylation of Mg_0.55_Al (60 °C) [8, 48].

**Fig. 4:**
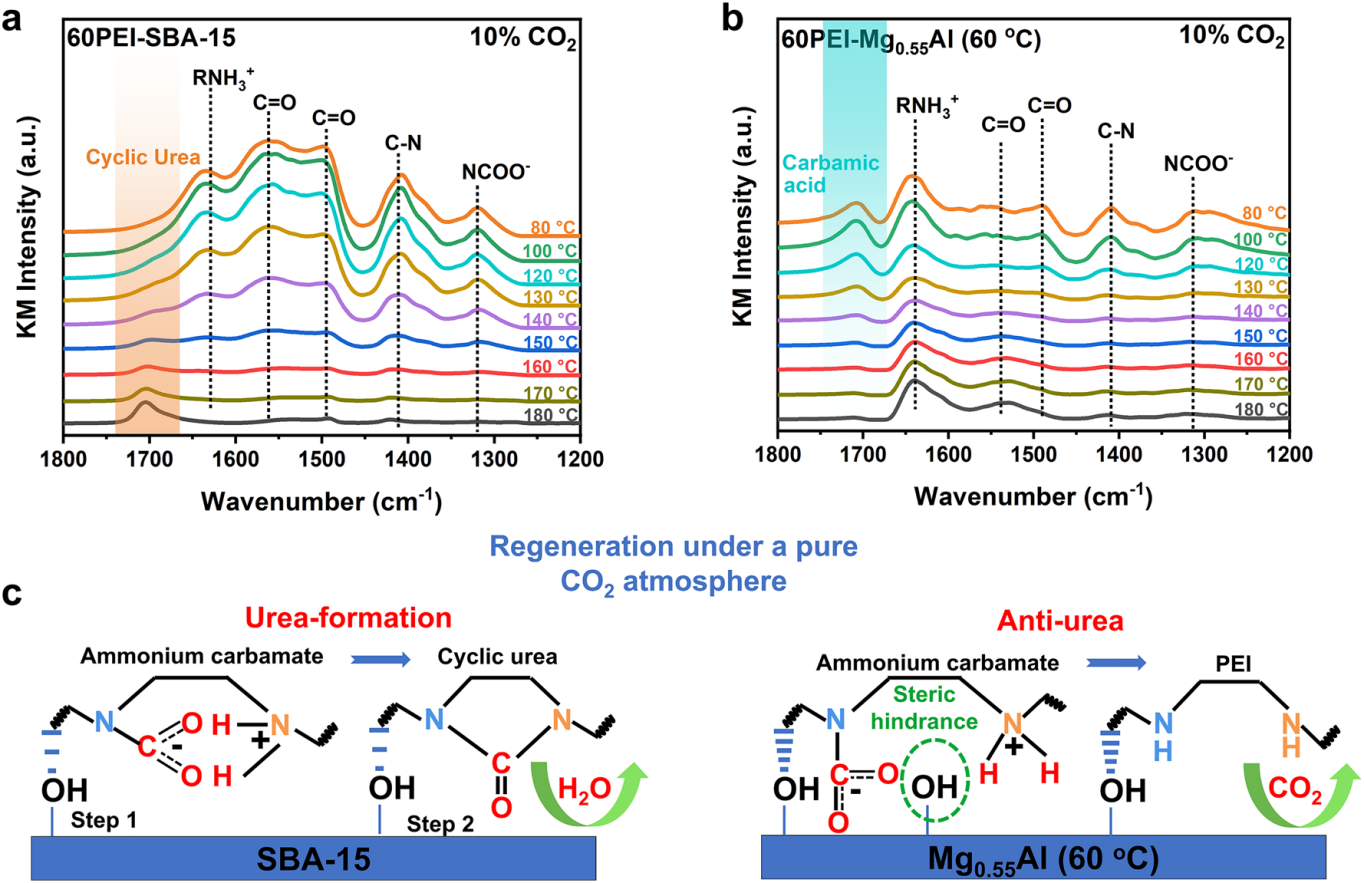
Effect of supports on the urea formation resistance of impregnated PEI. In situ DRIFTS spectra of passing 10% CO_2_ over the **a** 60PEI-SBA-15 and **b** 60PEI-Mg_0.55_Al (60 °C) samples while continuously increasing the temperature from 80 to 180 °C. **c** Illustration of the urea formation resistance of adsorbents under pure CO_2_ regeneration conditions

Figure 4a, b shows the in situ DRIFTS spectra of 60PEI-SBA-15 and 60PEI-Mg_0.55_Al (60 °C) exposed to 10% CO_2_ at different temperatures (80–180 °C). Over 60PEI-SBA-15, only carbamate species were observed in the 1300–1700 cm^−1^ range at 80 °C, due to the adsorption of CO_2_ [49]. Nevertheless, a newly developed infrared peak located at 1704 cm^−1^ assigned to the C=O stretching of cyclic urea emerged at 100 °C, and the intensity increased with the elevated temperature [50]. The intensity of the C=O stretching band reached its maximum at 180 °C, clearly confirming the pronounced formation of cyclic urea in the 60PEI-SBA-15 system. This explains the rapid decline in adsorption capacity of 60PEI-SBA-15 under a pure CO_2_ regeneration atmosphere (Fig. 1c). However, the formation of urea species was not appreciable over 60PEI-Mg_0.55_Al (60 °C), which only exhibited a weak stretching band at 1712 cm^−1^. In contrast, it demonstrated superior stability of CO_2_-bound species, with a prominent characteristic peak for carbamate species persisting even at 180 °C. The metal hydroxyl groups on Mg_0.55_Al (60 °C) can immobilize carbamate intermediates through strong hydrogen bonding, effectively preventing further dehydration of carbamates and thus inhibiting urea formation (Fig. 4c).

According to the findings above, we believe the nature of surface hydroxyl groups of support plays a key role in urea formation and oxidative degradation mechanisms. Theoretically, we can predict that PEI-Al_2_O_3_ and PEI-SiO_2_ should exhibit similar performance to PEI-Mg_0.55_Al (60 °C) and PEI-SBA-15. As shown in Fig. S8, the accumulation of urea products on PEI-SiO_2_ is markedly more pronounced than that on PEI-Al_2_O_3_. In terms of oxidative stability, both samples were pre-aged at 120 °C in a simulated flue gas comprising 10% CO_2_, 5% O_2_, and 3% H_2_O. The CO_2_ uptakes were evaluated at 75 °C with 10% CO_2_. Similar to PEI-Mg_0.55_Al (60 °C), PEI-Al_2_O_3_ exhibited significant oxidative degradation in the copresence of CO_2_ and O_2_. Its CO_2_ uptake sharply decreased from 1.80 mmol g^−1^ to 1.34, 0.93, and 0.59 mmol g^−1^ after aging for 5, 10, and 20 min, respectively. And similar to PEI-SBA-15, PEI-SiO_2_ demonstrated substantially better stability under the same conditions, maintaining a CO_2_ uptake of 1.73 mmol g^−1^ after aging for 20 min (Fig. S9). In all, we can conclude that PEI supported on Al–OH-containing substrates suffers from severe oxidative degradation during the CO_2_ capture step due to the breakage of amine-support hydrogen bonding networks, but exhibits enhanced anti-urea formation properties by preventing dehydration of carbamate products under a pure CO_2_ regeneration atmosphere. In contrast, the PEI supported on Si–OH-containing substrates exhibits an excellent anti-oxidative stability under simulated flue gas conditions by forming a robust hydrogen bonding protective network with Si–OH, but suffers from severe urea formation during the pure CO_2_ regeneration step.

### PEG-Induced Improvement Mechanism in Wet Flue Gases

Inspired by the hydroxyl group-dependent deactivation mechanisms for supported PEI adsorbents, we further incorporate a hydroxyl-containing additive PEG for improved performance. The aim is to assess whether PEG could (i) mitigate the oxidative degradation of PEI supported on Al–OH-containing substrates in the copresence of CO_2_ and O_2_, and (ii) address urea formation problem in PEI supported on Si–OH-containing substrates during pure CO_2_ regeneration.

The oxidative stability of 40PEI-20PEG-Mg_0.55_Al (60 °C) and 40PEI-20PEG-SBA-15 during CO_2_ adsorption/desorption cycles was systematically compared under conditions of 10% CO_2_ + 5% O_2_ for adsorption and pure N_2_ for desorption (Fig. S10). After 30 cycles, the CO_2_ uptake of 40PEI-20PEG-Mg_0.55_Al (60 °C) irreversibly decreased from 2.56 to 1.95 mmol g^−1^. This indicates that PEG incorporation could not effectively mitigate the oxidative degradation of PEI on Al–OH-containing substrates. In contrast, 40PEI-20PEG-SBA-15 exhibited enhanced oxidative stability under the same conditions, maintaining a CO_2_ uptake of 2.64 mmol g^−1^ after 30 cycles.

**Fig. 5:**
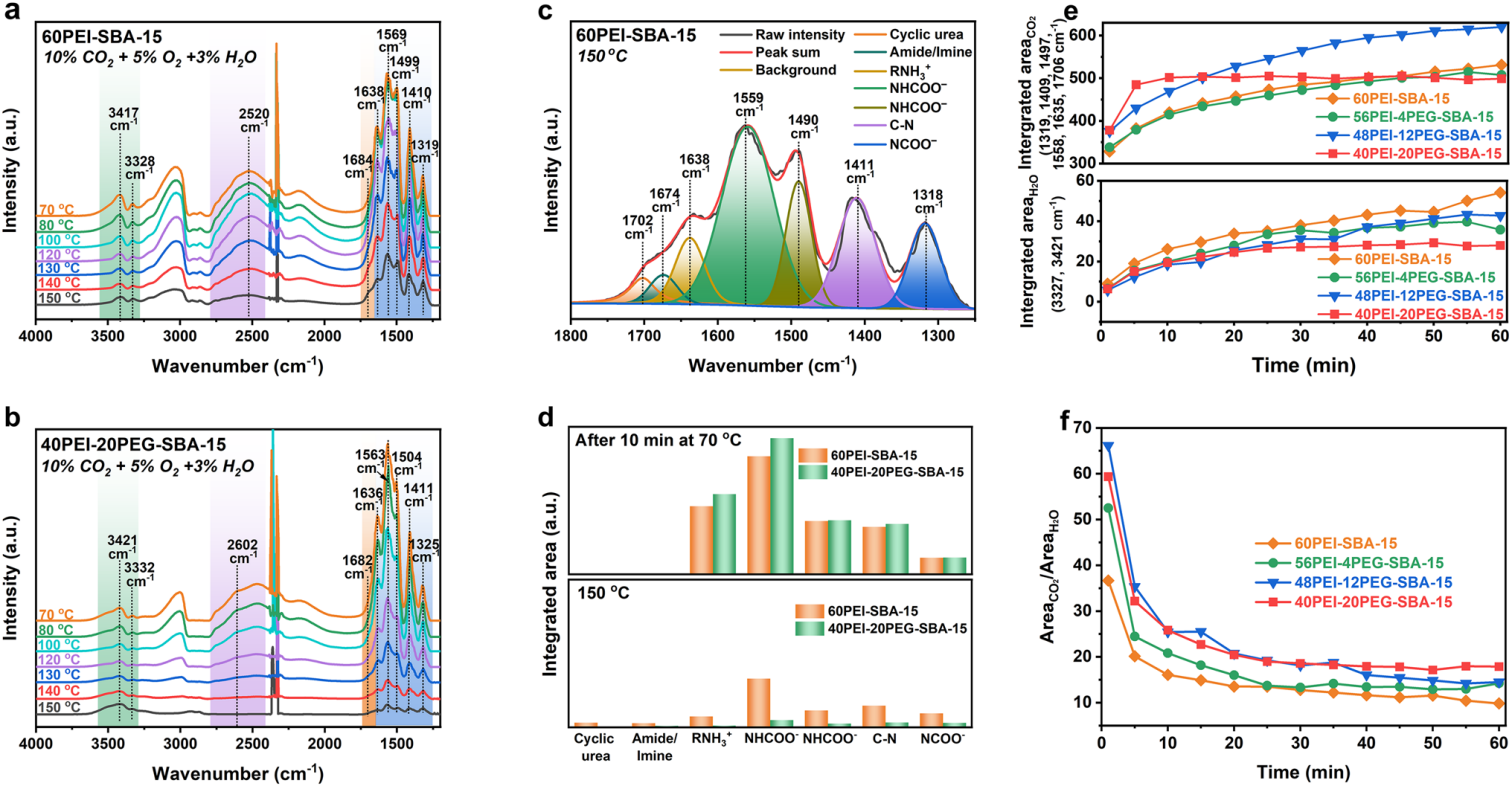
Improvement of PEG in wet flue gases. In situ DRIFTS spectra of passing 10% CO_2_ + 5% O_2_ + 3% H_2_O over **a** 60PEI-SBA-15 and **b** 40PEI-20PEG-SBA-15 while continuously increasing the temperature from 70 to 150 °C. **c** Deconvolution of spectra of 60PEI-SBA-15 obtained at 150 °C. **d** Integrated IR peak area recorded at 70 and 150 °C of passing 10% CO_2_ + 5% O_2_ + 3% H_2_O over the 60PEI-SBA-15 and 40PEI-20PEG-SBA-15 adsorbents. **e** Integrated IR peak for adsorbed CO_2_ and H_2_O and **f** the ratio of peak area recorded at 70 °C of passing 10% CO_2_ + 5% O_2_ + 3% H_2_O over the supported PEI/PEG adsorbents

Subsequently, the influence of PEG additive on the performance of PEI-SBA-15 adsorbents in simulated wet flue gas was thoroughly investigated using in situ DRIFTS (Fig. 5). Initially, both 60PEI-SBA-15 and 40PEI-20PEG-SBA-15 adsorbents were exposed to a gas stream comprising 10% CO_2_, 5% O_2_, and 3% H_2_O at 70 °C for 10 min, followed by heating from 70 to 150 °C at a rate of 10 °C min^−1^. Both samples exhibited the characteristic bands corresponding to adsorbed CO_2_ in the range of 1200–1800 cm^−1^. Figure 5c, d illustrates the deconvolution of the spectra (1200–1800 cm^−1^) and the integrated IR peak area corresponding to the bound CO_2_ species and amine degradation products. After a 10-min adsorption at 70 °C, more bound CO_2_ species were observed over 40PEI-20PEG-SBA-15, suggesting enhanced amine efficiency by PEG incorporation. Upon raising the temperature to 150 °C, much less bound CO_2_ species and almost negligible cyclic urea and oxidation products imine/amide were observed on 40PEI-20PEG-SBA-15 than 60PEI-SBA-15. These data confirm that the incorporation of PEG into PEI-SBA-15 could improve the CO_2_ desorption kinetics, anti-oxidation of supported PEI, and anti-urea formation in simulated wet flue gases.

The stabilization mechanism by PEG modification was then investigated by monitoring the formation of cyclic urea and amide/imide species over supported PEI/PEG adsorbents with different PEG loadings in 10% CO_2_ (Fig. S11) or pure O_2_ conditions (Fig. S12). With increase in PEG loading, the imine/amide species (1671 cm^−1^) resulting from oxidative degradation and the cyclic urea species (1705 cm^−1^) are gradually suppressed [21, 50]. The N 1*s* spectrum of PEI/SBA-15 adsorbents before and after PEG modification exhibits a slight shift to lower binding energies for the -NH_2_ and -NH- groups, suggesting an electron density transfer resulting from PEG-induced hydrogen bonding interactions [21, 24, 51]. Notably, the peak area proportion related to protonated amines gradually increases from 3.5% in 60PEI-SBA-15 to 3.6%, 3.8%, and 4.3% as the PEG loading increases from 4% to 20% (Fig. S13) [52–55]. This suggests that the abundant hydroxyl groups provided by PEG can result in the conversion of exposed free amines to protonated amines, which are less susceptible to degradation. In addition, the abundant hydrogen bonding between the aliphatic amines and the PEG hydroxyl groups can create a steric hindrance near the amine sites and thus retard the hydrolysis of carbamate species, effectively inhibiting urea formation. Unlike the strong hydrogen bonding network formed by the Al–OH groups of Mg_0.55_Al (60 °C), which immobilizes carbamate intermediates, the hydrogen bonding between PEG and PEI not only provides a protective effect but also enhances the regeneration efficiency of adsorbent, facilitating the rapid desorption of carbamate species and effectively mitigating urea formation. Although neat SBA-15 possesses similar hydroxyl groups to PEG, its amount is far from enough for the stabilization of carbamate species.

It has been reported that the inhibition of H_2_O co-adsorption is crucial for reducing regeneration heat [56]. In our study, we found that the adsorbed water observed in the range of 3290–3680 cm^−1^ and the hydronium ion observed in the range of 2400–2800 cm^−1^ over 40PEI-20PEG-SBA-15 were significantly less than those over 60PEI-SBA-15 (Fig. 5a, b) [49]. This result indicates that the modification of PEG could suppress the co-adsorption of H_2_O, which was further confirmed by monitoring the adsorption process at 70 °C in the presence of 10% CO_2_, 5% O_2_, and 3% H_2_O using in situ DRIFTS. The peaks associated with NCOO^–^ deformation vibration (1319 cm^−1^), C-N stretching vibration (1409 cm^−1^), C=O stretching vibration (1497 and 1558 cm^−1^), N–H deformation (1635 cm^−1^), and carbamic acid species (1706 cm^−1^) constitute the adsorbed CO_2_ species, while the O–H stretching bands of water molecules (3327 and 3421 cm^−1^) represent the adsorbed H_2_O species (Fig. S14) [40–42, 57]. These peaks are used to determine the integrated peak area, as shown in Fig. 5e, f. The reduction in H_2_O adsorption caused by PEG incorporation became more pronounced with the incorporation of CO_2_ and O_2_. The ratio of the peak areas of adsorbed CO_2_ to adsorbed H_2_O was determined. It was found that the PEI/SBA-15 system without PEG exhibited a large water adsorption capacity, with an Area_CO2_/Area_H2O_ ratio of 9.81 at 60 min. With the increase in PEG loading, the H_2_O adsorption capacity of PEI-PEG-SBA-15 samples decreased significantly. In particular, the ratio of Area_CO2_/Area_H2O_ reached 17.85 when the PEG loading was increased to 20 wt%. The PEG increases the overall length of amine polymer backbone through the formation of hydrogen bonding with amine centers. The alkyl chains of ethyl groups in PEG introduce additional steric hindrance, making it more difficult for water molecules to approach the amino groups and thereby imparting hydrophobicity to adsorbents. Furthermore, the introduction of PEG markedly accelerated the CO_2_ adsorption kinetics (Fig. 5e) [24]. The rapid interaction between CO_2_ and the amine sites led to the interlocking of amine chains, thereby impeding further interaction with H_2_O.

**Fig. 6:**
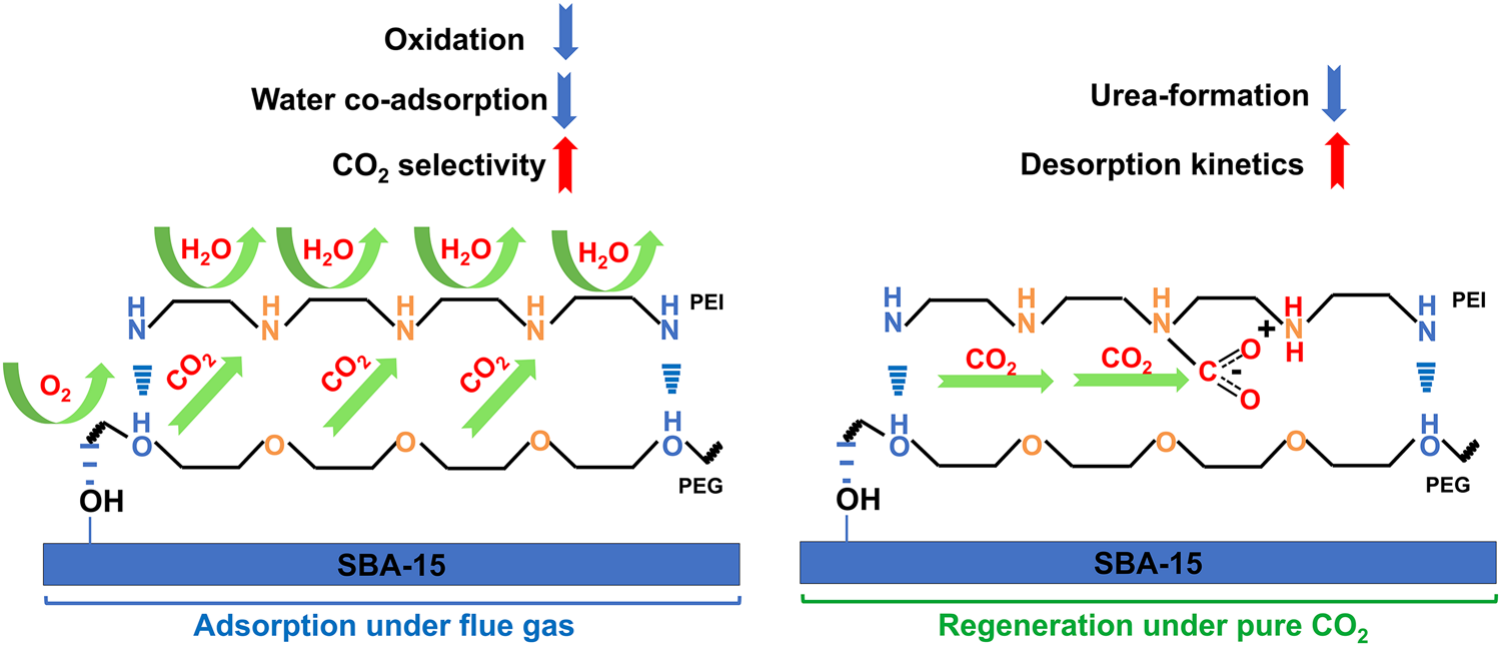
Illustration of the mechanism of PEG-induced improvements in wet flue gases. Improvement of PEG under CO_2_ capture from flue gas and pure CO_2_ regeneration conditions

In all, we demonstrated that the incorporation of PEG to PEI-SBA-15 improves its adsorption/desorption performance and cyclic stability. The hydroxyl groups of PEG play a key role in the mechanism, as illustrated in Fig. 6. The hydrogen bonding formed between the hydroxyl groups of PEG and the amine groups of PEI can enhance the oxidation resistance, reduce the H_2_O co-adsorption during the adsorption period, and significantly inhibit the urea formation and increase the CO_2_ desorption kinetics during the regeneration period.

### Performance of Ultra-Stable Adsorbent 40PEI-20PEG-SBA-15

**Fig. 7:**
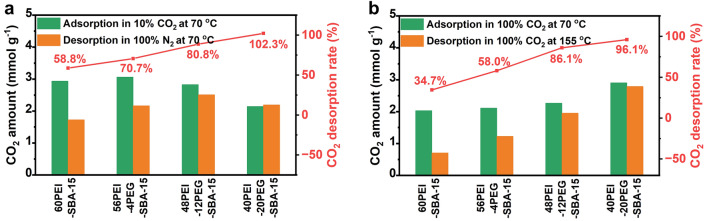
CO_2_ adsorption–desorption profiles of PEI-PEG-SBA-15 adsorbents. **a** Adsorption at 70 °C in 10% CO_2_ for 60 min and desorption at 70 °C in 100% N_2_ for 15 min and **b** adsorption at 70 °C in 100% CO_2_ for 10 min and desorption at 155 °C in 100% CO_2_ for 15 min

After a clear understanding of all involved deactivation processes and mechanisms of supported amines on different substrates, we designed an ultra-stable adsorbent system, which is particularly suitable for reversible flue gas CO_2_ capture. The CO_2_ adsorption–desorption behaviors as a function of PEG loading are presented in Fig. 7. It is apparent that the CO_2_ desorption efficiency of supported PEI improves with the increase of PEG loading. For the optimized 40PEI-20PEG-SBA-15 adsorbent, a complete regeneration can be achieved at 70 °C using an N_2_ purge. To yield highly concentrated CO_2_ streams suitable for the subsequent storage or utilization, the regeneration performance using pure CO_2_ as purge gas was further investigated. Aligned with the trend observed by N_2_ regeneration, the incorporation of PEG also led to a notable enhancement in desorption efficiency by CO_2_ regeneration at 155 °C. The regeneration efficiency was increased from 34.7% for 60PEI-SBA-15 to 96.1% for 40PEI-20PEG-SBA-15.

**Fig. 8:**
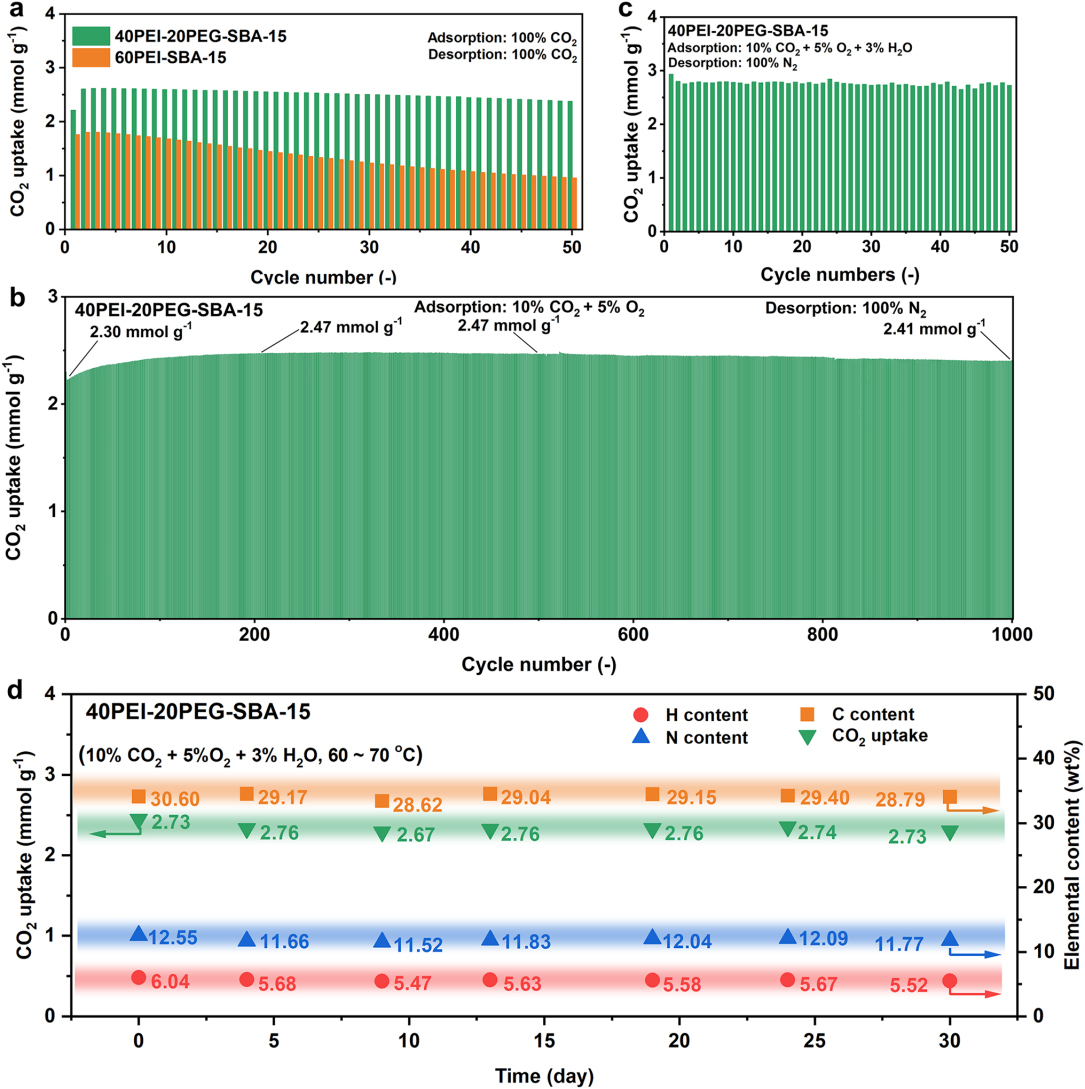
Lifetime studies of 40PEI-20PEG-SBA-15. **a** Uptake in 60PEI-SBA-15 and 40PEI-20PEG-SBA-15 adsorbents over 50 cycles of CO_2_ adsorption (at 70 °C in 100% CO_2_ for 2 min) and desorption (at 155 °C in 100% CO_2_ for 2 min). **b** Uptake in 40PEI-20PEG-SBA-15 adsorbents over 1000 cycles of CO_2_ adsorption (10% CO_2_ + 5% O_2_) and isothermal desorption at 70 °C (100% N_2_). **c** CO_2_ Uptake of 40PEI-20PEG-SBA-15 in the cyclic breakthrough tests (adsorption at 70 °C in 10% CO_2_ + 5% O_2_ + 3% H_2_O for 10 min and desorption at 70 °C in 100% N_2_ for 90 min). **d** CO_2_ uptake and C, H, and N content of 40PEI-20PEG-SBA-15 over one month on stream under 10% CO_2_ + 5% O_2_ + 3% H_2_O

The long-term cyclic stability of adsorbents was first investigated with pure CO_2_ as the purge gas for regeneration. As shown in Fig. 8a, the initial CO_2_ adsorption capacity of 60PEI-SBA-15 was 1.76 mmol g^−1^ in the first cycle. However, it rapidly decreased to only 0.96 mmol g^−1^ after 50 cycles, showing a pronounced deactivation. In contrast, 40PEI-20PEG-SBA-15 exhibited remarkable stability compared to 60PEI-SBA-15, with a final CO_2_ uptake of 2.38 mmol g^−1^, underscoring its excellent desorption performance as well as the superior resistance to urea formation-induced deactivation.

To further confirm its feasibility for commercial applications, we systematically investigated the reusability of 40PEI-20PEG-SBA-15 under 1000 consecutive adsorption–desorption cycles (Fig. 8b). The CO_2_ adsorption is performed in 10% CO_2_ + 5% O_2_ at 70 °C for 10 min, followed by regeneration in N_2_ purge gas at 70 °C for 15 min. The CO_2_ uptake of 40PEI-20PEG-SBA-15 showed a slight decrease of 0.07 mmol g^−1^ during the first two cycles due to some irreversible chemisorption, followed by a gradual increase from 2.23 to 2.47 mmol g^−1^ over the next 198 cycles. Subsequently, the working capacity became very stable at around 2.45 mmol g^−1^ and remained almost unchanged during the following 800 cycles. These results clearly demonstrated that the newly designed 40PEI-20PEG-SBA-15 adsorbent possesses both highly effective adsorption/desorption kinetics and remarkable long-term cyclic stability. We attribute the ultra-stable cyclic performance to the superior anti-oxidative property of PEI-SBA-15 itself during the adsorption period, and the improved desorption efficiency and anti-urea formation properties caused by PEG incorporation during the regeneration period.

It is well accepted that the water vapor in flue gas is always a challenge for CO_2_ capture; thus, the long-term stability in a more realistic gas condition (10% CO_2_ + 5% O_2_ + 3% H_2_O) was evaluated. The 40PEI-20PEG-SBA-15 adsorbent showed excellent cyclic stability, with an average CO_2_ uptake of 2.76 mmol g^−1^ over 50 cycles (Figs. 8c and S15). Notably, 40PEI-20PEG-SBA-15 with reduced amine loading exhibited a CO_2_ uptake of 2.94 mmol g^−1^, even higher than 60PEI-SBA-15 (2.85 mmol g^−1^) in the first cycle. This can be attributed to the PEG functionalization that enhances amine efficiency. Furthermore, the 40PEI-20PEG-SBA-15 adsorbent exhibited remarkable stability during a one-month continuous aging experiment conducted in a simulated flue gas flow (10% CO_2_ + 5% O_2_ + 3% H_2_O, 60–70 °C), with no obvious loss of N and H species detected (Fig. 8d). In conclusion, the optimized 40PEI-20PEG-SBA-15 adsorbent possesses high CO_2_ uptake, fast adsorption/desorption kinetics, and superior chemical stability, rendering them highly attractive for reversible flue gas CO_2_ capture.

## Conclusions

In this study, we investigated the intrinsic deactivation mechanisms of two adsorbents PEI-Mg_0.55_Al and PEI-SBA-15 for reversible CO_2_ capture from flue gases under various conditions. We revealed that the nature of surface hydroxyl groups on different supports is the key parameter that influences the stability. With the Mg_0.55_Al hydroxide support containing metal-OH groups, the supported PEI exhibits better oxidative stability under pure O_2_ due to the stronger hydrogen bonding interaction between Mg/Al–OH and amines. However, the PEI-Mg_0.55_Al faces severe oxidative degradation with the copresence of CO_2_ and O_2_, which involves the breakage of amine-support hydrogen bonding networks. Furthermore, under pure CO_2_ regeneration conditions, the metal hydroxyl Al–OH on the Mg_0.55_Al (60 °C) stabilized carbamate intermediates through robust hydrogen bonding, effectively preventing further dehydration and demonstrating excellent resistance to urea formation. In contrast, with the SBA-15 support containing nonmetal hydroxyl Si–OH groups, the supported PEI could be easily oxidized by pure O_2_ due to the weak hydrogen bonding interaction between Si–OH and amines. While in the copresence of CO_2_ and O_2_, this hydrogen bonding network becomes more stable and is less susceptible to disruption by CO_2_. Moreover, this hydrogen bonding network enhances the flexibility of the PEI chains, facilitating a more rapid reaction with CO_2_ to form carbamate species, which effectively inhibits the oxidative attack. Nevertheless, during pure CO_2_ regeneration, PEI supported on Si–OH-containing substrates remained prone to significant urea formation. Further, we reveal that the incorporation of PEG does not mitigate the oxidative deactivation of the Al–OH-containing support in the presence of both CO_2_ and O_2_; however, it markedly enhances the resistance of PEI supported on Si–OH-containing substrates to urea formation. The hydrogen bonding formed between the hydroxyl groups of PEG and the amine groups of PEI plays the key role, not only providing protective effects by converting exposed free amines into more stable protonated amines but also facilitating the rapid desorption of carbamate species, thereby effectively mitigating urea formation. The alkyl chains of ethyl groups in PEG also increase the hydrophobicity of the adsorbent and effectively reduce the co-adsorption of H_2_O. Based on the intrinsic understanding of the degradation mechanisms, we successfully designed and prepared an ultra-stable adsorbent 40PEI-20PEG-SBA-15 that demonstrates outstanding stability and retention of a remarkable CO_2_ capacity of 2.45 mmol g^−1^ over 1000 adsorption–desorption cycles, along with negligible capacity loss during aging in simulated flue gas (10% CO_2_ + 5% O_2_ + 3% H_2_O) for one month at 60–70 °C. Considering the potential irreversible poisoning of solid amine adsorbents by acid gases (e.g., NO_*x*_/SO_2_) in the flue gas mix, which could also impact long-term stability [8], further studies are recommended to investigate the hydroxyl group-dependent stability mechanisms of these adsorbents in the presence of NO_*x*_/SO_2_. Overall, this work represents a profound comprehension of the hydroxyl group-dependent deactivation mechanisms, thereby furnishing a substantial theoretical foundation for the prospective design of commercially viable amine-containing flue gas CO_2_ adsorbents.

## Supplementary Information

Below is the link to the electronic supplementary material.

Supplementary file1 (DOCX 2555 kb)
