# Supplementary material for: Design of Ultra-Stable Solid Amine Adsorbents and Mechanisms of Hydroxyl Group-Dependent Deactivation for Reversible CO2 Capture from Flue Gas
Source: Nanomicro Lett. 2025 Feb 28;17:170. doi: 10.1007/s40820-025-01664-w (PMC11871205; doi:10.1007/s40820-025-01664-w)
Supplement: Supplementary file 1 — Supplementary file1 (DOCX 2555 kb) [file 40820_2025_1664_MOESM1_ESM.docx]

Supporting Information for

**Design of Ultra-Stable Solid Amine Adsorbents and Mechanisms of Hydroxyl Group-Dependent Deactivation for Reversible CO_2_ Capture from Flue Gas**

Meng Zhao^1,2^, Liang Huang^1,2^, Yanshan Gao^1,2^*, Ziling Wang^1,2^, Shuyu Liang^1,2^, Xuancan Zhu^3^, Qiang Wang^1,2^*, Hong He^4^, Dermot O’Hare^5^*

^1^College of Environmental Science and Engineering, Beijing Forestry University; Beijing 100083, P. R. China

^2^State Key Laboratory of Efficient Production of Forest Resources, Beijing Forestry University; Beijing 100083, P. R. China

^3^Research Center of Solar Power & Refrigeration, Institute of Refrigeration and Cryogenics, Shanghai Jiao Tong University, Shanghai 200240, P. R. China

^4^State Key Joint Laboratory of Environment Simulation and Pollution Control, Research Centre for Eco-Environmental Sciences, Chinese Academy of Sciences; Beijing 100085, P. R. China

^5^Chemistry Research Laboratory, Department of Chemistry, University of Oxford, Mansfield Road, Oxford OX1 3TA, United Kingdom

*Corresponding authors. E-mail: [yanshan_gao@bjfu.edu.cn](mailto:yanshan_gao@bjfu.edu.cn) (Yanshan Gao); [qiangwang@bjfu.edu.cn](mailto:qiangwang@bjfu.edu.cn) (Qiang Wang); [dermot.ohare@chem.ox.ac.uk](mailto:dermot.ohare@chem.ox.ac.uk) (Dermot O’Hare)

**Supplementary Figures and Tables**


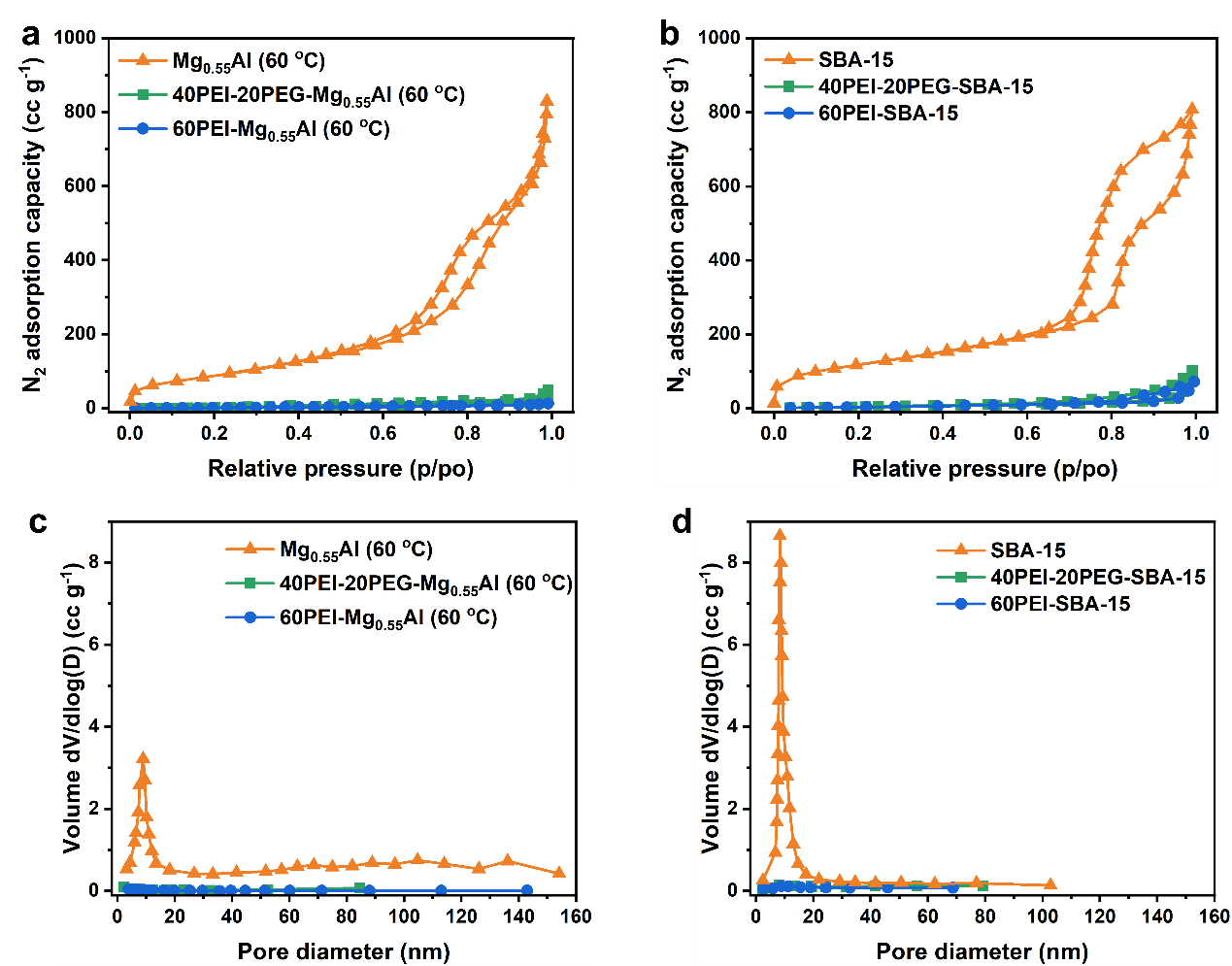


**Fig. S1** N_2_ isotherms for the pristine and PEI/PEG-functionalized **a** Mg_0.55_Al (60 ^o^C) and **b** SBA-15 samples. Pore distributions for the pristine and PEI/PEG-functionalized **c** Mg_0.55_Al (60 ^o^C) and **d** SBA-15 samples

**
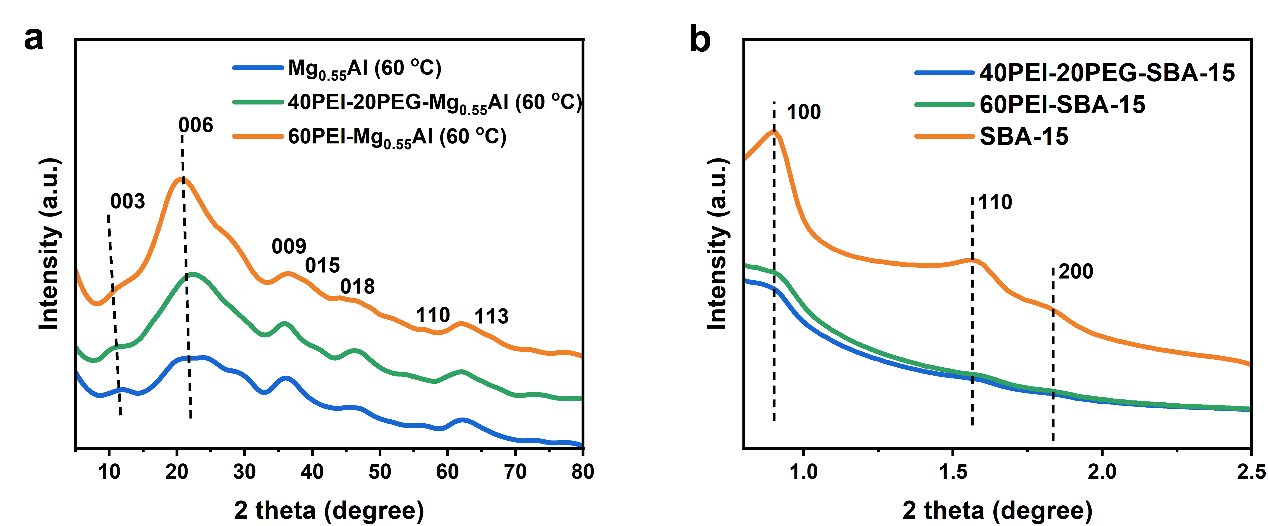
Fig. S2** XRD patterns of the pristine and PEI/PEG-functionalized **a** Mg_0.55_Al (60 ^o^C) and **b** SBA-15 samples


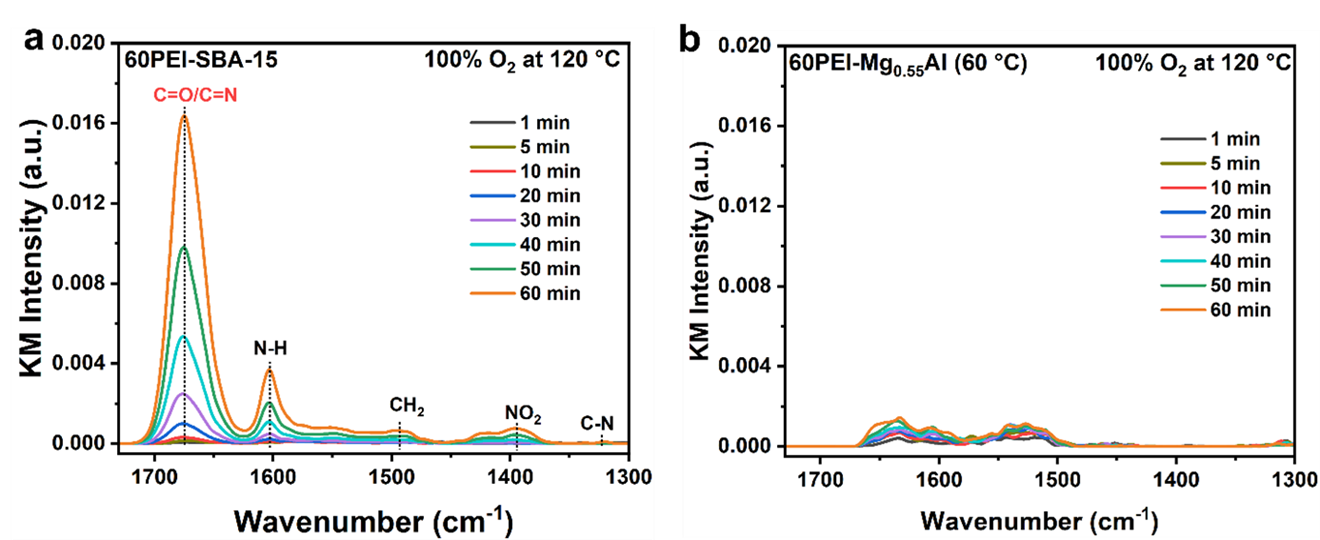


Fig. S3 In-situ DRIFTS spectra recorded at 120 ^o^C of passing pure O_2_ over the a 60PEI-SBA-15 and b 60PEI-Mg_0.55_Al (60 °C)


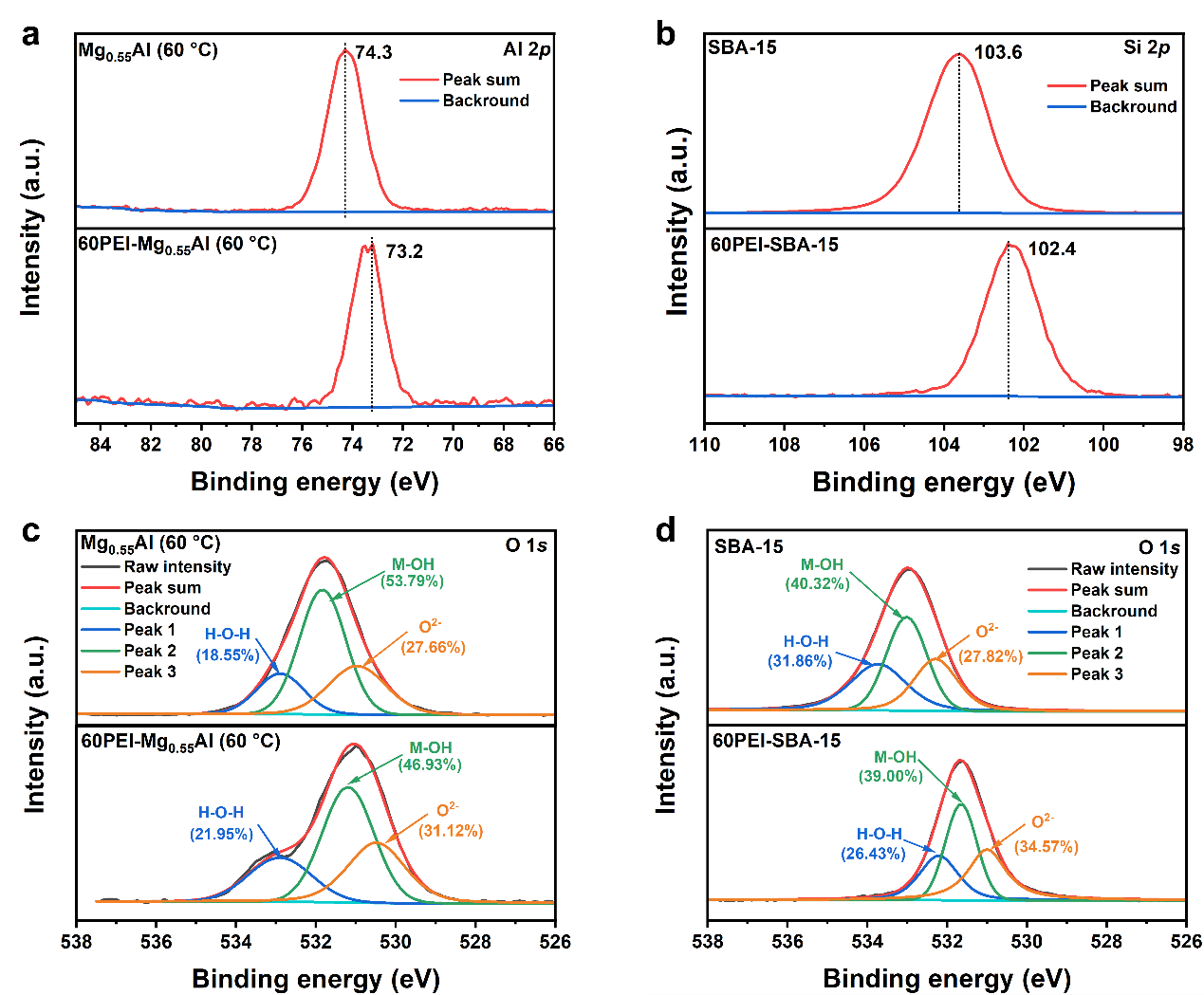


Fig. S4 XPS spectra of a Al 2p for Mg_0.55_Al (60 °C), b Si 2p for SBA-15, and c, d O 1s for Mg_0.55_Al (60 °C) and SBA-15, respectively, before and after 60 wt% PEI impregnation


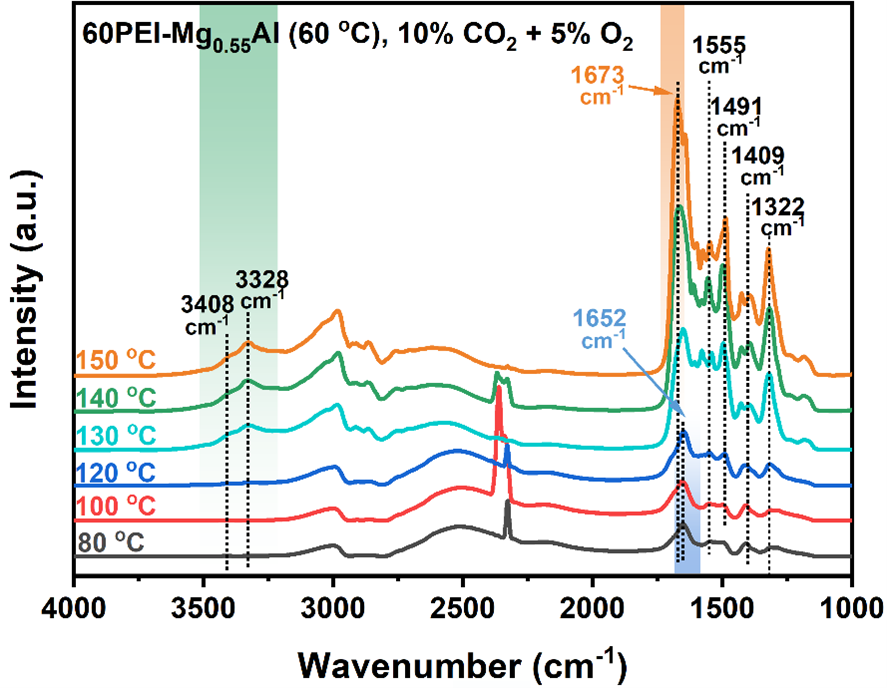


Fig. S5 In-situ DRIFTS spectra of passing 10% CO_2_ + 5% O_2_ over the 60PEI-Mg_0.55_Al (60 °C) sample while continuously increasing the temperature from 80 °C to 150 °C


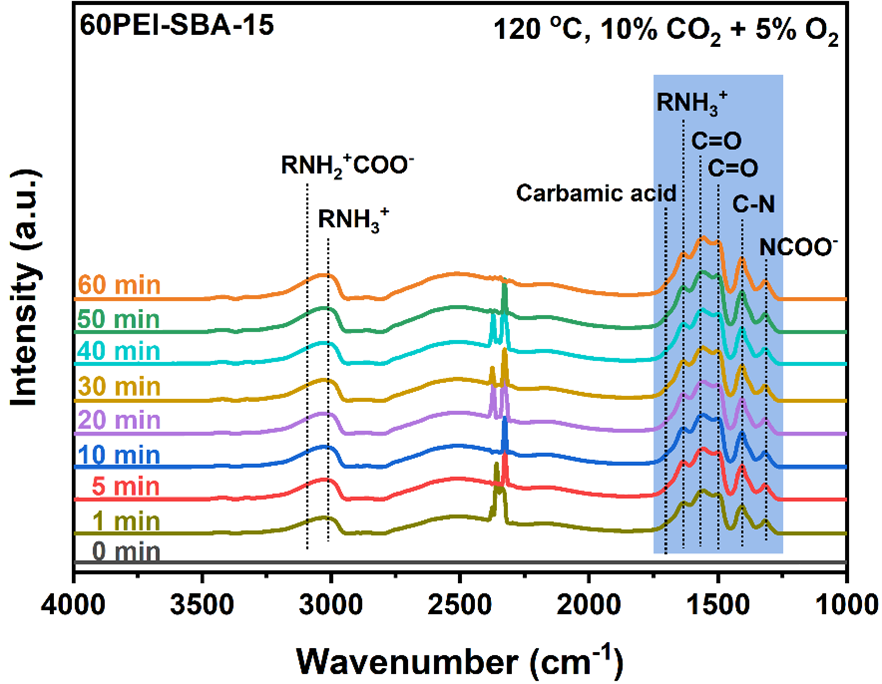


Fig. S6 In-situ DRIFTS spectra recorded at 120 ^o^C of passing 10% CO_2_ + 5% O_2_ over the 60PEI-SBA-15 adsorbents


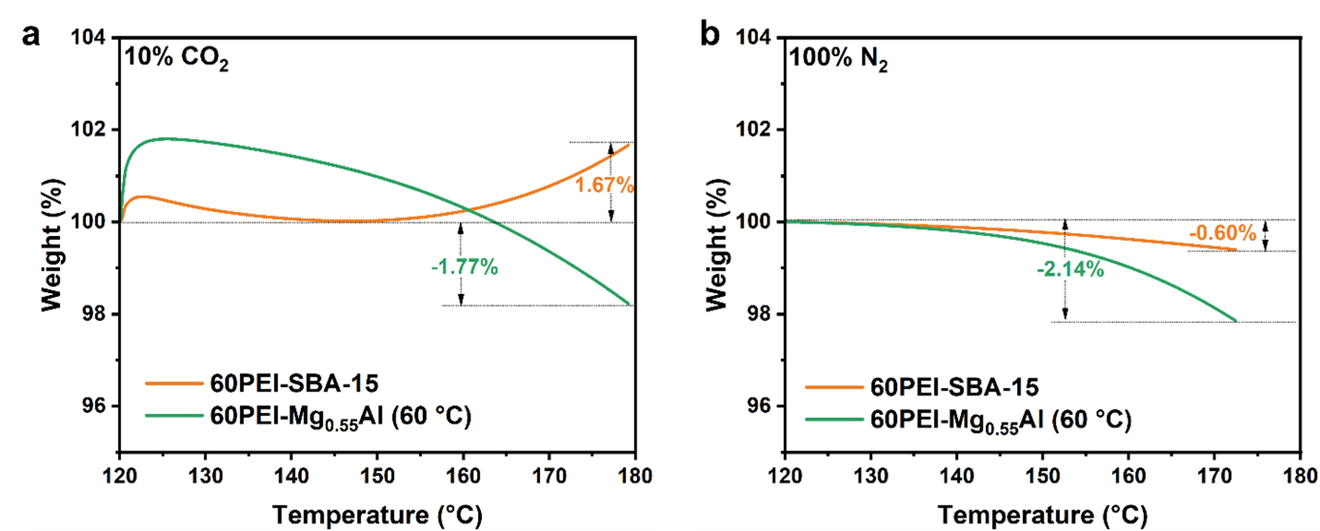


Fig. S7 TG curves of passing a 10% CO_2_, and b 100% N_2_ over the 60PEI-Mg_0.55_Al (60 °C) and 60PEI-SBA-15 samples


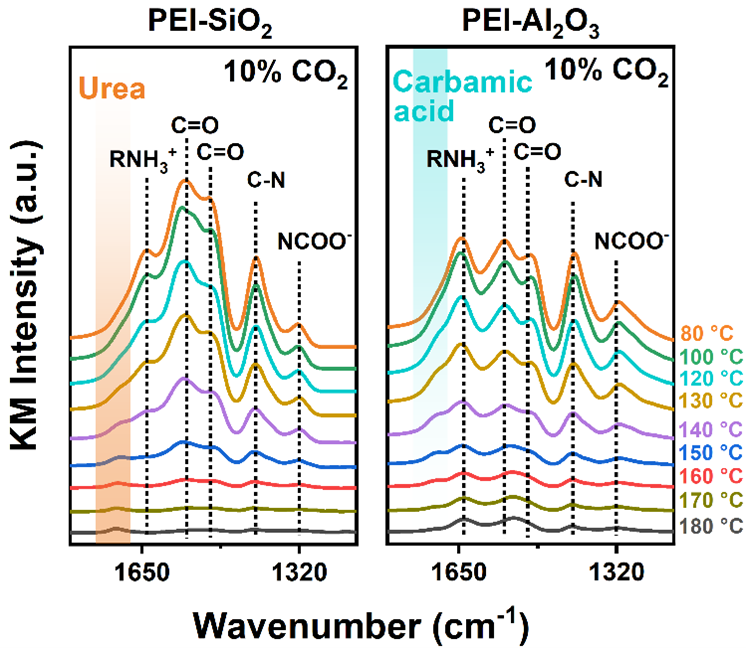


Fig. S8 In-situ DRIFTS spectra of passing 10% CO_2_ over the 33PEI-SiO_2_ and 33PEI-Al_2_O_3_


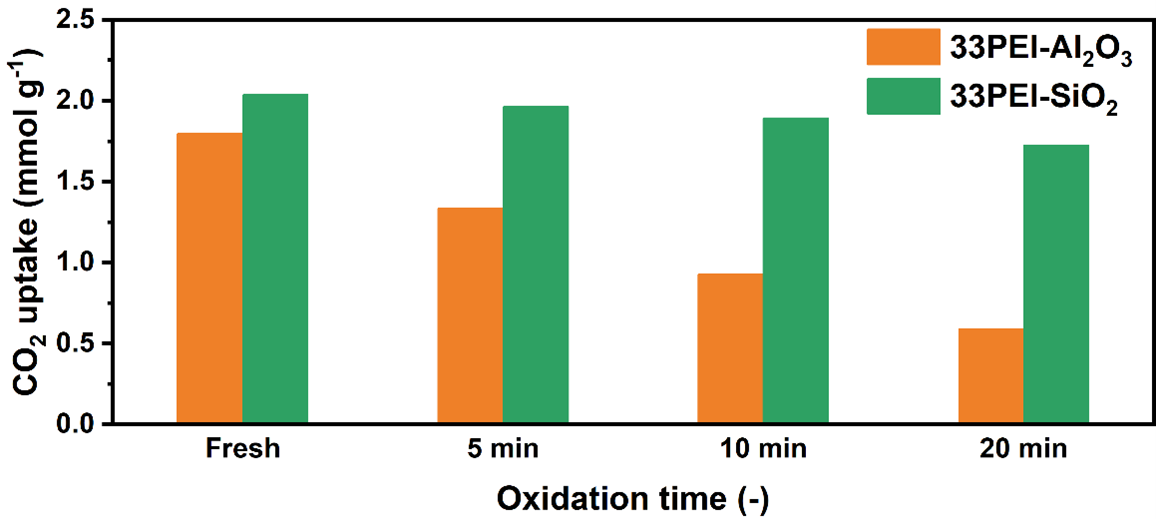


Fig. S9 CO_2_ uptakes at 75 °C under 10% CO_2_ for 33PEI-Al_2_O_3_ and 33PEI-SiO_2_ before and after aging at 120 °C in 10% CO_2_ + 5% O_2_ + 3% H_2_O


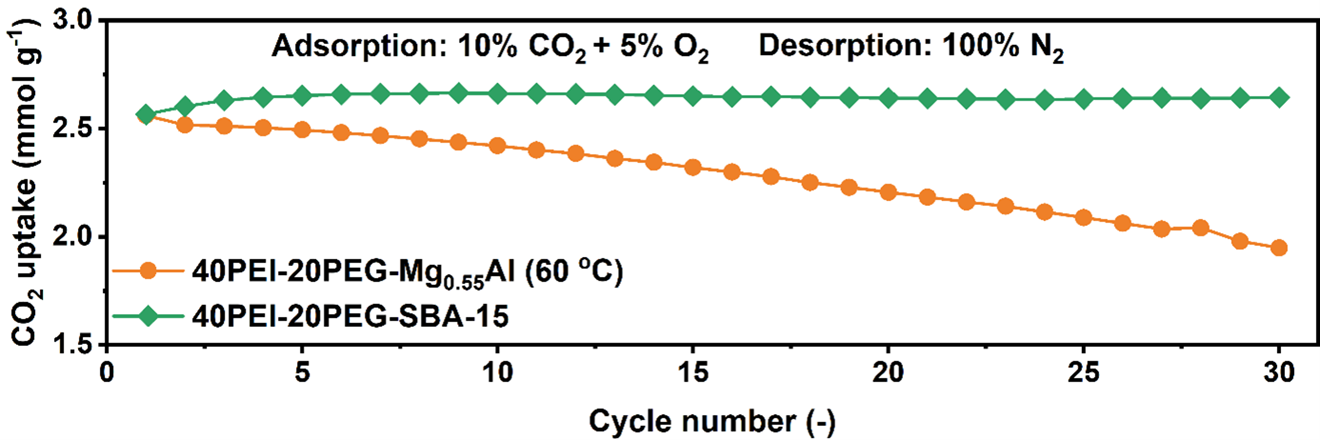


Fig. S10 CO_2_ uptakes of 40PEI-20PEG-Mg_0.55_Al (60 °C) and 40PEI-20PEG-SBA-15 over 30 cycles (adsorption at 75 °C in 10% CO_2_ + 5% O_2_ for 10 min and desorption at 120 °C in 100% N_2_ for 15 min


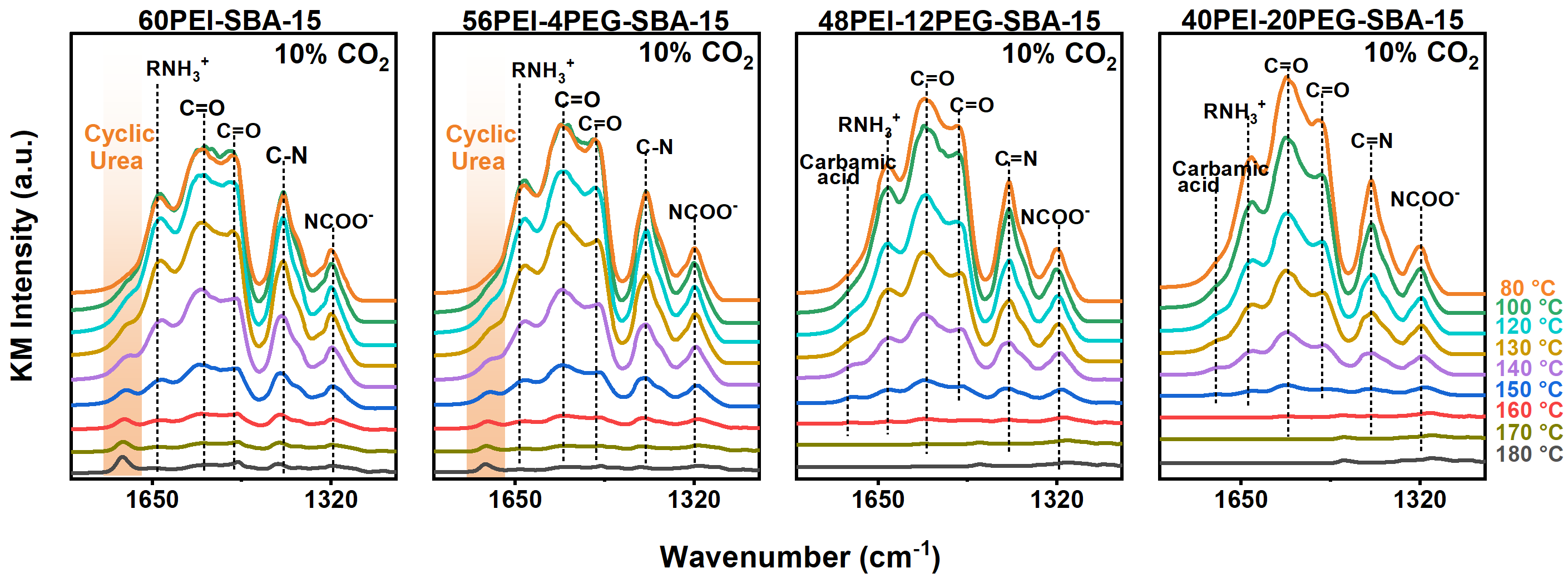


Fig. S11 In-situ DRIFTS spectra of passing 10% CO_2_ over the supported PEI/PEG adsorbents while continuously increasing the temperature from 80 to 180 °C


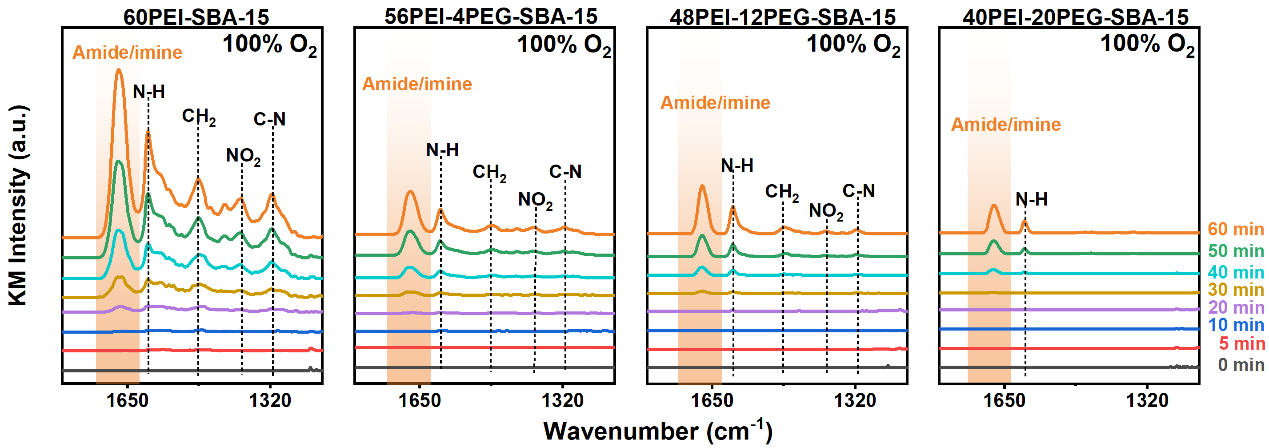


Fig. S12 In-situ DRIFTS spectra recorded at 120 °C of passing 100% O_2_ over the supported PEI/PEG adsorbents


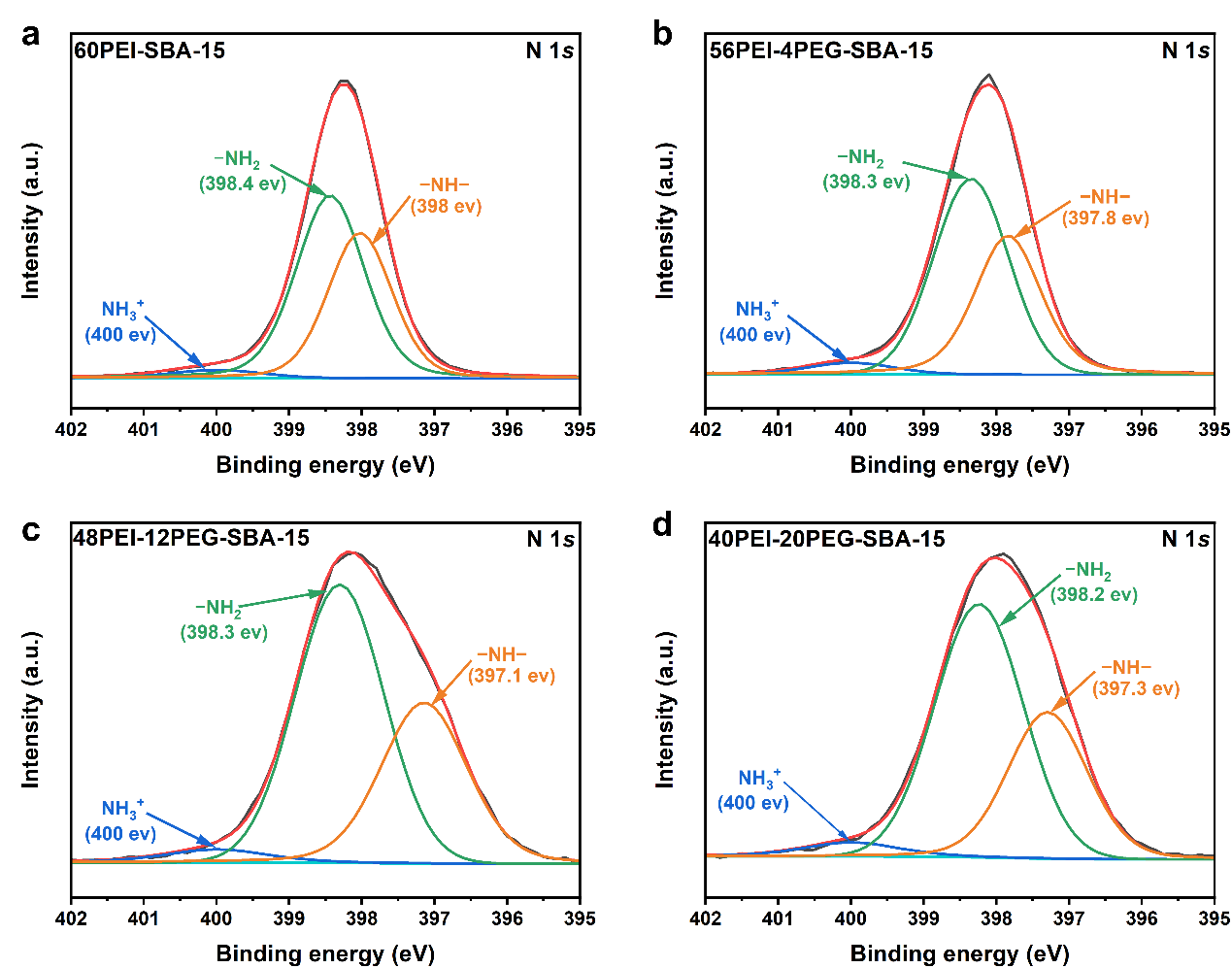


Fig. S13 XPS spectra of N 1s of a 60PEI-SBA-15, b 56PEI-4PEG-SBA-15, c 48PEI-12PEG-SBA-15, and d 40PEI-20PEG-SBA-15 sample


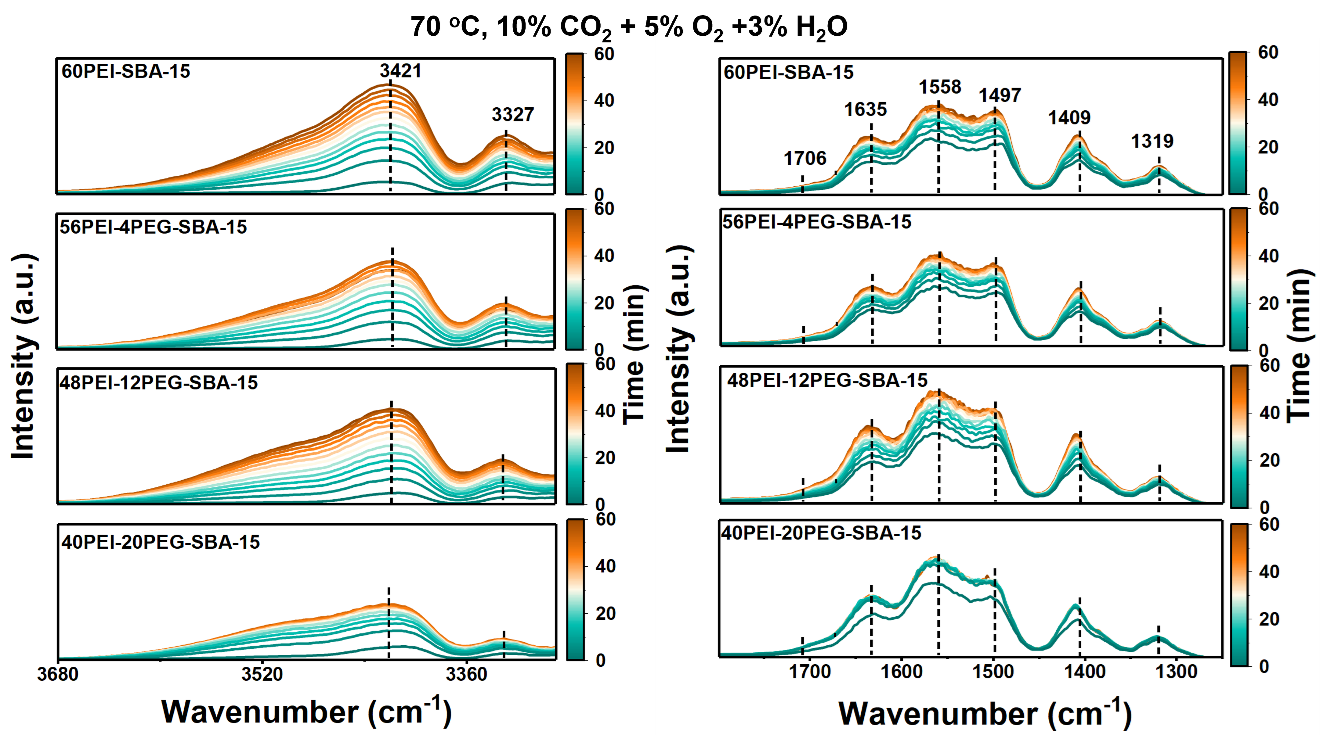


Fig. S14 In-situ DRIFTS spectra recorded at 70 °C of passing 10% CO_2_ + 5% O_2_ + 3 vol% H_2_O over the supported PEI/PEG adsorbents


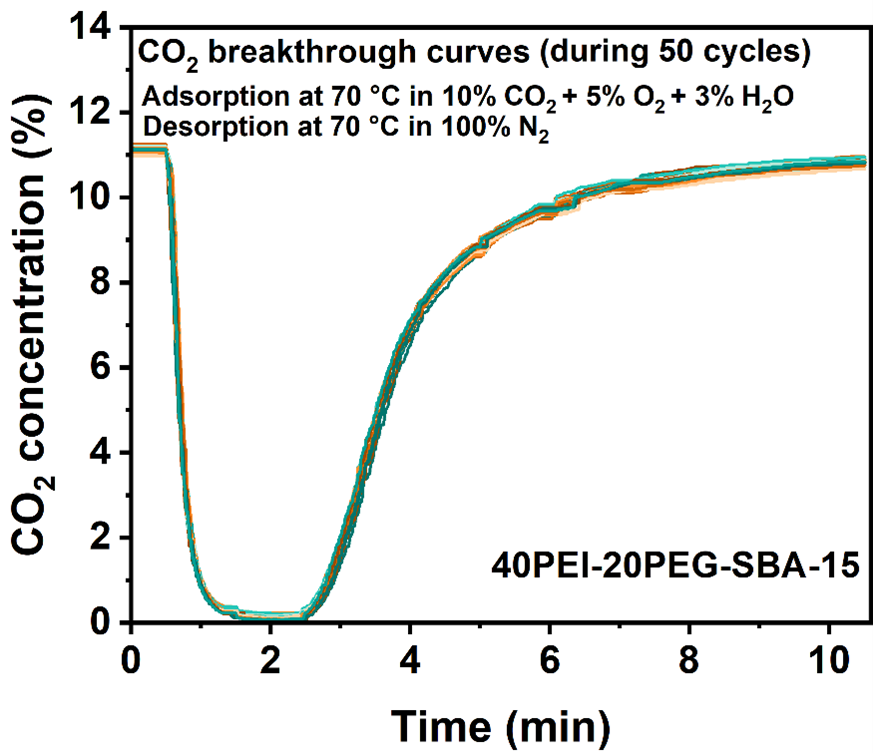


Fig. S15 CO_2_ breakthrough curves of 40PEI-20PEG-SBA-15

Table S1 Textural properties of the pristine and PEI/PEG-functionalized Mg_0.55_Al (60 °C) samples

| **Sample** | **Specific**  **surface area**  **(m^2^ g^-1^)** | **Average**  **pore size**  **(cc g^-1^)** | **Average pore volume (nm)** | |
| --- | --- | --- | --- | --- |
| Mg_0.55_Al (60 **°C**) | 328.1 | 1.50349 | | 7.96 |
| 60PEI-Mg_0.55_Al (60 **°C**) | 6.1622 | 0.027186 | | 6.28 |
| 40PEI-20PEG-  Mg_0.55_Al (60 **°C**) | 8.5496 | 0.08757 | | 17.79 |

**Table S2** Textural properties of the pristine and PEI/PEG-functionalized SBA-15 samples

| **Sample** | **Specific**  **surface area**  **(m^2^ g^-1^)** | **Average**  **pore size**  **(cc g^-1^)** | **Average pore volume (nm)** | |
| --- | --- | --- | --- | --- |
| SBA-15 | 420.0883 | 1.430324 | | 5.95 |
| 60PEI-SBA-15 | 16.5764 | 0.123793 | | 13.43 |
| 40PEI-20PEG-SBA-15 | 19.123 | 0.167791 | | 16.4 |
